# Hybrid SnO_2_/TiO_2_ Nanocomposites for Selective Detection of Ultra-Low Hydrogen Sulfide Concentrations in Complex Backgrounds

**DOI:** 10.3390/s16091373

**Published:** 2016-08-27

**Authors:** Alexander Larin, Phillip C. Womble, Vladimir Dobrokhotov

**Affiliations:** 1 VAON LLC, KY, USA, Bowling Green, KY 42101, USA; alexander.larin@bgfky.com (A.L.); phillip.womble@bgfky.com (P.C.W.); 2 Applied Physics Institute, Western Kentucky University, Bowling Green, KY 42101, USA

**Keywords:** sensor, micro-electromechanical systems (MEMS), hydrogen sulfide, nanocomposites

## Abstract

In this paper, we present a chemiresistive metal oxide (MOX) sensor for detection of hydrogen sulfide. Compared to the previous reports, the overall sensor performance was improved in multiple characteristics, including: sensitivity, selectivity, stability, activation time, response time, recovery time, and activation temperature. The superior sensor performance was attributed to the utilization of hybrid SnO_2_/TiO_2_ oxides as interactive catalytic layers deposited using a magnetron radio frequency (RF) sputtering technique. The unique advantage of the RF sputtering for sensor fabrication is the ability to create ultra-thin films with precise control of geometry, morphology and chemical composition of the product of synthesis. Chemiresistive films down to several nanometers can be fabricated as sensing elements. The RF sputtering technique was found to be very robust for bilayer and multilayer oxide structure fabrication. The geometry, morphology, chemical composition and electronic structure of interactive layers were evaluated in relation to their gas sensing performance, using scanning electron microscopy (SEM), X-ray diffraction technique (XRD), atomic force microscopy (AFM), Energy Dispersive X-ray Spectroscopy (EDAX), UV visible spectroscopy, and Kelvin probe measurements. A sensor based on multilayer SnO_2_/TiO_2_ catalytic layer with 10% vol. content of TiO_2_ demonstrated the best gas sensing performance in all characteristics. Based on the pattern relating material’s characteristics to gas sensing performance, the optimization strategy for hydrogen sulfide sensor fabrication was suggested.

## 1. Introduction

Hydrogen sulfide is a hazardous gas with strong odor. Lower concentrations of it can cause nausea, vomiting and eye irritation. Higher concentrations of hydrogen sulfide can lead to shock, convulsions, inability to breathe, and coma. It is extremely important to detect hydrogen sulfide leaks at early stages of exposure in order to prevent poisoning. Detection of hydrogen sulfide plays an important role in oil and gas exploration and production.

Some of the major H_2_S monitoring technologies include electrochemical, infrared and metal oxide sensors. Recently developed physisorptive gas sensor technology is based on charge transfer in two-dimensional tin disulfide at low operating temperatures [1]. Gas sensors based on electrochemical and MOX gas sensing technologies hold the largest market share of around 21% and 20%, respectively. Better efficiency, fast response time, and cost effectiveness are some important factors that led to the growth of gas sensors based on these technologies. Some of the major players engaged in gas sensors manufacturing include City Technology, Alphasense, Membrapor, Dynament, Figaro Engineering, and others. At the same time, state-of-the-art electrochemical and infrared sensor technologies already met their technical limitations. In the last decade there was a very limited change in design, physical dimensions, power consumption or the cost of these sensors. Their performance in terms of sensitivity, selectivity, stability or a lifetime have not been significantly improved either.

Metal oxide (MOX) sensors are widely used for hydrogen sulfide monitoring [2,3,4,5,6,7]. For more than two decades now, small and simple gas sensors have been commercially available. Typically, they are manufactured by the sol-gel method, in which metal oxide layers are deposited in the form of viscous paste and then baked in the inert environment, creating thick films. Hydrogen sulfide MOX sensors from Figaro (TGS sensors) and Henan Hanwei Electronics Co., Ltd. (Henan, China) (MQ sensors) are manufactured using this method. Most of the commercial metal oxide sensors utilize purely macroscopic solutions and their dimensions are comparable with infrared and electrochemical sensors. At the same time, the novel innovative nanotechnology and MEMS-based solutions are currently available primarily in the area of metal oxide sensors.

Recently, a great deal of effort has been focused on utilizing nanostructures for sensor applications. Significant advantages of nanomaterials as sensitive elements arise from their extremely high surface-to-volume ratio, which makes their electromechanical and thermal properties strongly dependent on surface phenomena. The condition of the surface of the nanostructure is determined by the parameters of the surrounding medium, which allows one to establish a direct correspondence between the properties of the nanostructure and the parameters of the medium in which this nanostructure is immersed. This basic principle drives the sensing mechanisms of most nanomaterials. Kaneti et al. [8] conducted experimental and theoretical studies of ethanol sensing by Au and Pd decorated tin oxide flower-like nanorods. It was found that deposition of Au and Pd nanoparticles on the surface of the SnO_2_ nanorods is advantageous in: enhancing the sensitivity towards ethanol (9–15 times); reducing the response/recovery time (by 15–40 s), and significantly decreasing the optimum operating temperature (from 250 to 175 °C). Tang et al. [9] studied 1-D assembly of binary nanoparticles as gas sensors. Binary 1-D nanowires consisting of both SnO_2_ nanoparticles and Au nanorods, fabricated through a “substrate–particle solution template” assembling method, demonstrated highly enhanced gas sensitivity toward acetone under ambient conditions. Yue et al. [10] conducted a density functional theory study of adsorption of the OH Group on SnO_2_ Oxygen Bridges. The authors analyzed factors influencing gas sensing performance of SnO_2_ sensors. The adsorption of water, oxygen, ethanol, and acetic acid onto the SnO_2_ (110) surface was studied using molecular dynamics (MD) simulations.

Various nanomaterials demonstrate high sensitivity toward hydrogen sulfide: nanocrystals, nanofibers, nanowires, nanosprings, nanoribbons, etc. [2,6,7,11]. However, their thermal and electrical stability over an extended period of time in complex backgrounds is lacking. Additionally, reproducibility of nanomaterials-based sensors is limited, which complicates the scalable manufacturing techniques and commercial mass production of this type of sensors. In contrast to nanomaterials, metal oxide thin films are very stable and reproducible, but limited in sensitivity. The current effort in sensor technology is concentrated on development of devices combining the properties of nanomaterials and thin films, which are very sensitive and selective and at the same time stable and reproducible.

There is an interest toward developing a high-quality MOX sensor for detection of H_2_S. Tin oxide (SnO_2_) is one of the most common chemiresistive materials due to its high catalytic activity toward a broad range of analytes [2,5,12]. It has been found that certain semiconductor additives improve the performance of tin oxide layers in terms of sensitivity and selectivity, compared to bare tin oxide. It was reported that grain boundaries between different oxides have higher catalytic activity, compared to single-oxide grain boundaries [13,14,15,16,17]. Recently, SnO_2_-based metal oxide structures with n-p and n-n heterojunction have attracted a great deal of interest as gas sensing materials [2,13,14,15,16,17,18,19].

The concept of electron transport through the grain boundary from material with lower work function to material with higher work function with the formation of contact potential in the equilibrium state has been used to describe the electron distribution at the heterojunction [20]. The role of heterojunctions in molecular adsorption and catalytic reactions was considered by Yamazoe et al. [14]. In this work, the authors proposed how the contact potential at the interface of different oxides affects the electron transport through the contact. It was also suggested that the heterojunction resistance at the interface of two different oxides is more sensitive to the local gas environment than the contact resistance at the interface of two grains of the same nature. This means that the content of the multilayer oxide structure can be optimized in order to achieve maximum sensing performance.

Enhanced detection of H_2_S has been reported by using different heterojunction structures SnO_2_/CuO, SnO_2_/WO_3_ and SnO_2_/ZnO [3,4,18,21]. The sensitivity of multilayer oxides was found to be close to the sensitivity of novel nanomaterials, but at the same time the thin film sensors demonstrated high stability and repeatability. Among other advantages of utilization of multilayer oxides in chemiresistors are low activation temperature, short activation time, short time of response and short time of recovery.

One of the challenges for hydrogen sulfide detection by metal oxides is sulfur poisoning. Many sensors lose their catalytic activity over time. TiO_2_ catalyst and catalyst support is known for its high sulfur resistance [22,23,24,25]. It is one of the catalysts that are widely used in industry for H_2_S decomposition and oxidation, also known as the Claus process. Even though TiO_2_ is one of the most interactive with H_2_S catalysts, it is a challenge to use it as a sensing element due to its very low electrical conductivity. At the same time, excellent thermal, mechanical, and catalytic properties makes it a good candidate for doping material [26,27,28,29,30,31]. The results obtained by various research groups on detection of H_2_S by using metal oxides are summarized in Table 1.

There are several well-known deposition methods for sensing element fabrication: sol-gel method, screen printing, thermal evaporation, nanocasting etc. [32,33,34,35,36,37,38] RF magnetron sputtering technique is a well-known method for thin film fabrication. The unique advantage of the RF sputtering for sensor fabrication is the ability to create ultra-thin films with precise control over geometry, morphology and chemical composition of the product of synthesis. Chemiresistive films down to several nanometers can be fabricated as sensing elements. The RF sputtering technique was found to be very robust for bilayer and multilayer oxide structure fabrication. We will call a bilayer structure a thin film obtained by a consecutive deposition of two oxides. We will call a multilayer structure a thin film obtained by multiple consecutive depositions of two oxides.

In the present work, novel multilayer and bilayer SnO_2_/TiO_2_ structures were evaluated for H_2_S detection. The effect of SnO_2_/TiO_2_ n-n heterojunction on sensing performance was studied. Thickness, morphology, and activation temperature of the sensing element were optimized for maximum sensor response. Sensitivity, selectivity, stability, response and recovery time as well as the resistance to sulfur poisoning superior to the previously published data and commercial analogs were achieved as a result of this study.

## 2. Experimental Section

### 2.1. Fabrication and Characterization of a Suspended Microheater Platform

**Figure 1:**
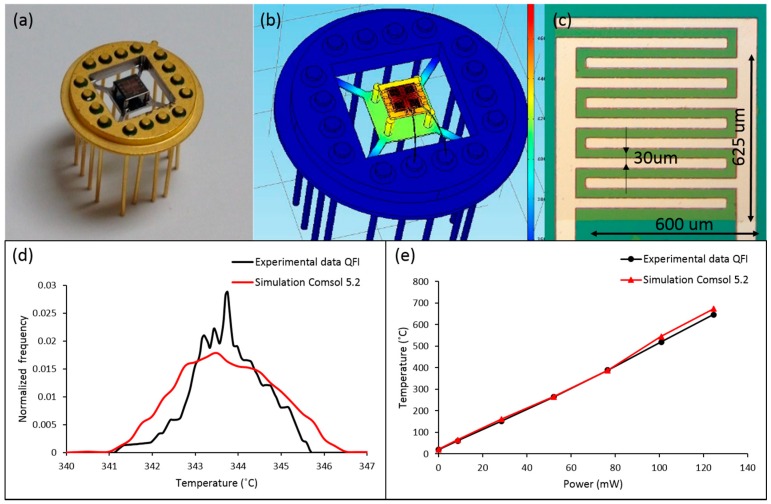
(**a**) Simulated temperature distribution over the microplatform attached to a TO package; (**b**) optical images of the sensor platform attached to a modified TO package; (**c**) a single sensing element area; (**d**) simulated and experimental temperature distribution across the sensing area under 70 mW heating power; (**e**) simulated and experimental data of the sensor average temperature over different power dissipation 8–120 mW.

A MEMS-based suspended membrane with a cross-shaped heating element and interdigitated sensor’s electrodes was fabricated for precise temperature control over the interactive metal oxide layer (Figure 1). 

The microplatform contains four identical square sensor elements in the corners and a cross-shaped heater in the center of the platform. The hot plate was cleanroom fabricated by a multi-step procedure: photolithography, sputtering deposition, liftoff, back side photolithography, reactive ion etching, deep reactive ion etching, and dicing. The heating element and interdigitated sensor’s electrodes were made out of 300 nm platinum deposited by magnetron sputtering (PVD 75 Lesker). A 5 nm buffer layer of titanium was deposited prior to platinum deposition for better adhesion. The membrane structure was fabricated by Deep Reactive Ion Etching (DRIE). The membrane thickness 50 µm was verified by surface profilometer KLA-Tencor Alpha-Step IQ. The surface temperature on the suspended membrane was first simulated using Comsol 5.2 software (COMSOL Inc., Stockholm, Sweden) and then compared with the experimental data from Quantum Focus Instruments (QFI) thermal imaging system, which is able to capture thermal images of the platform featuring 0.1 °C temperature and 5 µm spatial resolutions. The microplatform temperature profiles were obtained for different values of power dissipation across the heating element. It was also found that the temperature gradient along the sensing element area does not exceed 5 °C when the average surface temperature is above 350 °C. The microplatform was developed for four identical sensors in order to obtain small statistics over sensor response and stability characteristics. Although in the present study the sensitive elements on the platform are identical, the future intent is to expand the scope of this research by depositing different interactive layers on the same platform.

### 2.2. Oxides Deposition and Characterization

**Figure 2:**
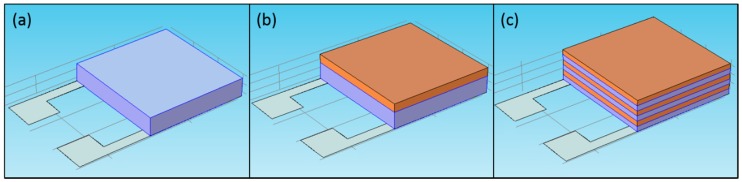
The schematics of a single-layer (**a**); a bilayer (**b**); and a multilayer (**c**).

Thin films of TiO_2_, SnO_2_, SnO_2_/TiO_2_ multilayer structure and SnO_2_/TiO_2_ bilayer structure were deposited by RF magnetron sputtering using SnO_2_ and TiO_2_ three inch targets with purity of 99.99% and 99.998%, respectively. The sample rotation speed during the deposition was set up to 30 rpm for equal thickness distribution across the sample area. The deposition was conducted at room temperature and no special bias voltage was applied to the wafer. The schematics of a single-layer, a bilayer and a multilayer are shown in Figure 2a–c, respectively. The single-oxide samples (SnO_2_, TiO_2_) were sputtered under 12 mTorr of argon (Ar) pressure and RF power 200 W. The bilayer samples of TiO_2_/SnO_2_ were fabricated during the two step process: main layer deposition (SnO_2_) and surface doping layer deposition (TiO_2_). The multilayer TiO_2_/SnO_2_ structures were prepared by multiple consequent sputtering of two metal oxides. Different volume contents of TiO_2_ in SnO_2_ were obtained by varying power of the TiO_2_ source during its deposition phase. The multilayer structure was constructed out of a total of 6 layers: 3 layers of SnO_2_ and 3 layers of TiO_2_. After the deposition, the total thickness of each sample was verified by surface contact profilometer (KLA-Tencor 500 Alpha-Step IQ, (TENCOR Instruments, Mountain View, CA, USA). During the sample preparation eight different sample structures were prepared (Table 2). After the deposition, all the samples were annealed in a tube furnace (MKS OTF 1200x, MTI Corporaion, Richmond, CA, USA) under 500 °C for 48 h in ultra-zero grade air (UZ300 Airgas 100 sccm).

The crystal structure of samples was evaluated by X-ray diffraction method (XRD). The XRD spectrum of samples was collected by a Thermo ARL (model XTRA, Thermo Fisher Scientific Waltham, MA, USA) X-ray diffraction machine (Cu Kα radiation wavelength was 0.15056 nm). A scanning electron microscope (SEM Zeiss Supra 35, Carl Zeiss AG, Oberkochen, Germany) was utilized to study the surface microstructure of the samples. The chemical composition of the samples was obtained by Energy Dispersive X-ray Spectroscopy (EDAX) analysis. The band gap and work function of tin oxide and titanium oxide were evaluated by UV visible spectrometer AvaSpec-ULS2048L-EVO (Avantes, Louisville, CO, USA) and Kelvin probe measurement, respectively.

### 2.3. Gas Delivery System and Data Acquisition

**Figure 3:**
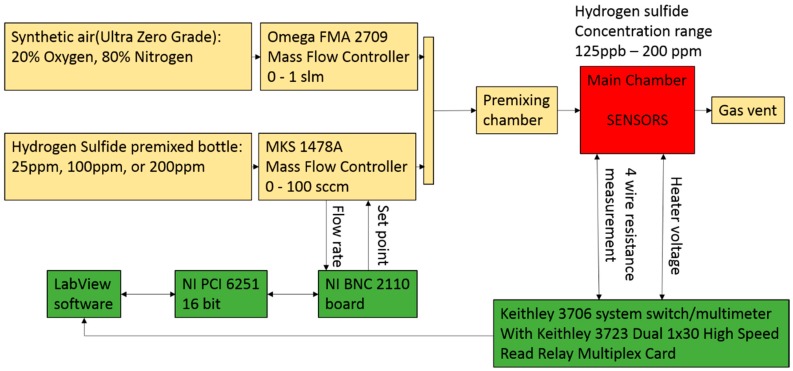
Gas delivery and data collection system.

The schematic of gas delivery and data acquisition system is shown in Figure 3. Sensor response characterization was conducted in a small (1 cm^3^) environmental chamber. The total flow rate through the chamber was fixed at 100 sccm during the experiment. All the data was collected under atmospheric pressure. The sensor resistance was measured with the Keithley 3706 system (Keithley Instruments, Cleveland, OH, USA) switch/multimeter connected to a PC through Labview interface. The resistance of the sensor was measured with a sample rate of 10 Hz. A system of mass flow controllers (Omega GMA 2709 and MKS 1478A, OMEGA Engineering, INC., Stanford, CT, USA) interfaced with Labview through PCI NI 6251 and BNC21110 was utilized to produce specific concentrations of the target gas. The gas delivery sistem was able to produce different concentrations of hydrogen sulfide in a range from 125 ppb to 200 ppm. The exposure time was chosen to be 4 min. During this time, the sensor signal was able to reach the saturation point for a particular concentration. After exposure, the chamber was flashed with 100 sccm of clean dry sinthetic air until the sensor signal returned to its original baseline. The sensor response was defined as S = R_Air_/R_Gas_, where R_Air_ is the sensor resistance in clean dry air and R_Gas_ is the sensor resistance upon exposure to hydrogen sulfide.

## 3. Results and Discussion

After the deposition, samples were annealed at 500 °C in the tubular furnace for 48 h. The choice of platinum as a material for electrodes and heater fabrication is crucial for preservation of the device stability under annealing. It was chosen for its exceptional chemical resistance in combination with high mechanical, electrical and thermal stability over a wide range of temperatures. The melting point of platinum (1768.3 °C) is much higher than the average annealing temperature (300–900 °C) for most of the metal oxides. Platinum electrodes can also be used for extreme annealing temperatures 1000–1200 °C, if necessary.

**Figure 4:**
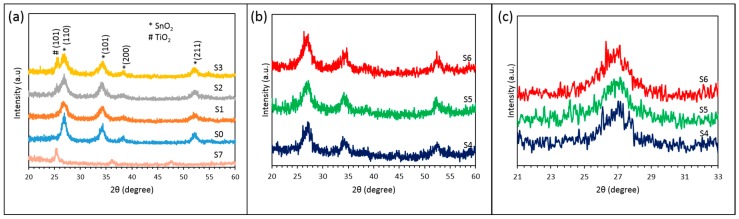
XRD spectroscopy of the (**a**) samples S0–S3 and S7; (**b**) samples S4–S6; and (**c**) zoom in on the major peaks of the samples S4–S6.

After the annealing, a nanocrystalline structure of oxides was observed by X-ray diffraction (XRD). The XRD patterns were recorded at a scanning rate of 1.2 times per second and a scanning step size of 0.02°. The XRD patterns were received from larger (1 × 1 inch) glass samples. These samples were prepared simultaneously with sensor fabrication. The simultaneous and uniform coating of several samples in a single run is one of the advantages of the reactive ion sputtering technique. A continuous rotation of the sample holder stage during the deposition process assures a uniform coating over the entire group of samples. The scanning range for all the samples was from 20° to 60°. Figure 4 shows the XRD pattern of all the samples (S0–S7). The XRD spectrum of pure SnO_2_ (S0) showed strong diffraction peaks at 2θ = 26.92°, 34.22°, 38.22°, and 52.17° corresponding to (110), (101), (200) and (211) crystal faces of rutile structure of SnO_2_ [20]. The anatase structure of TiO_2_ was identified by the major diffraction peak at 2θ = 25.43° [39]. The XRD analysis of the bilayer samples S1, S2 and S3 showed diffraction peaks similar to SnO_2_ crystal structure and additional peak at 2θ = 25.4° corresponding to (101) crystal faces of anatase structure of TiO_2_ was detected for samples S2 and S3. The XRD analysis of the composite SnO_2_/TiO_2_ structure (S4–S6) revealed three major peaks similar to SnO_2_ (S0). The position of the major diffraction peak of multilayer oxides shifts slightly from 2θ = 26.78° (S6) to 2θ = 26.87° (S5) and 2θ = 26.91° (S4) with decreasing % vol. of TiO_2_. In addition, the average crystal size of all the samples based on the major diffraction peak was calculated by using the Scherrer formula (Equation (1)):

(1) $$D=\frac{K\lambda }{\beta \cos\left(\theta \right)}$$

where *D* is the average size of nanocrystals, *λ* is the X-ray wavelength (1.5056 nm), *β* is the line broadening at half the maximum peak intensity (FWHM), *K* = 0.9 is a dimensionless shape factor and θ is the major diffraction peak position. The average size of SnO_2_ (S0) nanocrystals after the annealing process was found to be *d* = 7.87 nm. The characteristic size of nanocrystals for a multilayer SnO_2_/TiO_2_ structure was found to be smaller, compared to pure SnO_2_: *d* = 4.87 nm (S4), *d* = 4.54 nm (S5) and *d* = 4.09 nm (S6). The crystal size of the TiO_2_ (S7) was calculated to be 4.21 nm. The smaller grain size of the composite oxides (S4–S6) could be an advantage for gas sensing properties. During the XRD analysis, samples S4–S6 showed no specific peaks correlated to TiO_2_ crystal structure. However, a noticeable asymmetry, as well as a slight shift in the major peak of the multilayer structure in Figure 4c, may be attributed to the overlap of TiO_2_ and SnO_2_ peaks, caused by the small TiO_2_ nanocrystals present in the layer.

**Figure 5:**
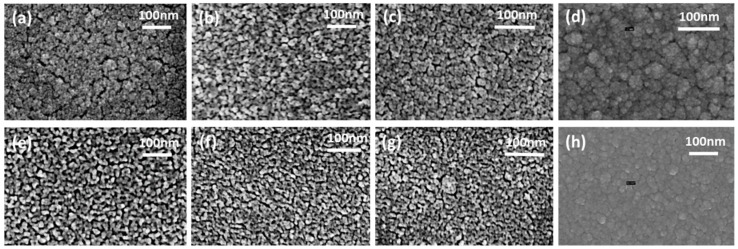
SEM images of the crystalline structure of samples S0–S7: (**a**) SnO_2_ (S0); (**b**) SnO_2_/TiO_2_ bilayer structure 30 nm + 5 nm (S1); (**c**) SnO_2_/TiO_2_ bilayer structure 30 nm + 10 nm (S2); (**d**) SnO_2_/TiO_2_ bilayer structure 30 nm + 20 nm (S3); (**e**) SnO_2_/TiO_2_ multilayer 5% of TiO_2_ (S4); (**f**) SnO_2_/TiO_2_ multilayer 20% of TiO_2_ (S5); (**g**) SnO_2_/TiO_2_ multilayer 50% of TiO_2_ (S6); (**h**) TiO_2_ (S7).

The morphology of samples S0–S7 was also studied by SEM (Zeiss Supra 35), as shown in Figure 5. All the samples demonstrated rough and porous polycrystalline structure with short neck-like interconnections between the grains. It can be seen that the porosity of the samples S0–S3 was gradually decreasing with the increasing content of TiO_2_. The SEM software analysis was used to determine the average grain size of the samples S0–S7. The grain size of the pure SnO_2_ (S0) and SnO_2_/TiO_2_ (S1–S3) bilayer structures from SEM analysis were found to be in a range of 10–15 nm.

The SnO_2_/TiO_2_ (S4–S6) multilayer structure also showed a slight decrease in porosity with increasing of TiO_2_ content from 5% vol. to 20% vol. The grain size of the multilayer SnO_2_/TiO_2_ structures (S4–S6) was in the range of 5–10 nm. It was found that sample S4 has more uniform grain size distribution and higher porosity in comparison to the rest of the samples. The combination of small grains with high porosity of samples S4, S5 and S6 creates favorable conditions for catalytic reactions thanks to the large surface area and high number of active sites.

### Sensor Performance Characteristics

**Figure 6:**
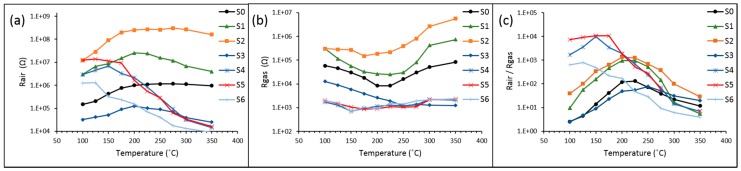
(**a**) Sensors resistance in air under different temperature conditions 100–350 °C; (**b**) resistance of the sensors in the presence of 10 ppm of H_2_S as a function of temperature; and (**c**) various responses of sensors toward 10 ppm of H_2_S over the temperature range.

The sensors’ (S0–S7) performance characteristics were first investigated over a wide temperature range of 100–350 °C for exposures to 10 ppm of H_2_S in synthetic air (Figure 6). The optimized temperature conditions for H_2_S detection were found for each sensor S0–S6 (Table 3). The pure TiO_2_ (S7) sample did not show any noticeable response to 10 ppm of H_2_S over the temperature range 100–350 °C. The bilayer SnO_2_/TiO_2_ structure (S2) demonstrated a much higher response of 1.88 × 10^3^ to 10 ppm to H_2_S gas at lower temperature of 200 °C compared to pure SnO_2_ (S0) sensitivity of 1.31 × 10^2^ at 225 °C. The highest sensor response to 10 ppm of H_2_S of 1.06 × 10^4^ was observed for SnO_2_/TiO_2_ (S5) composite structure at an even lower temperature of 150 °C. It was demonstrated that SnO_2_/TiO_2_ multilayer material has superior sensitivity toward H_2_S at lower temperatures. The sensor performance characteristics of the SnO_2_ based sensor with 10% vol. of TiO_2_ (S5) was found to be more efficient compared to the other results from previous reports (Table 1).

The content of TiO_2_ in the hybrid structure was found to be a crucial parameter that determines sensor performance. Both types of hybrid oxide structures (bilayer and multilayer) demonstrated decline in sensitivity for high contents of TiO_2_ in the layer. A thick (20 nm) compact layer of TiO_2_ deposited over the SnO_2_ layer (S3) affected the layer porosity and caused a decrease in sensor response due to the lack of SnO_2_ surface exposure to ambient air even at a higher temperature (300 °C). The bilayer structure of SnO_2_ with 20 nm of TiO_2_ coating demonstrated very low resistance over the temperature range 100–350 °C, compared to the other bilayer samples with thinner TiO_2_ coating. Increasing content of TiO_2_ within the multilayer structure (S6) from 10% to 20% decreased the sensitivity of the sensor. The sensor S6 with 20% vol. of TiO_2_ demonstrated low resistance over the temperature range 100–350 °C and lower sensitivity compared to the other complex oxides with lower TiO_2_ concentration.

Sensors S2 (bilayer) and S5 (multilayer) demonstrated the highest sensitivity to H_2_S in their groups due to the optimized content of TiO_2_. The bilayer sensor (S2) demonstrated the highest resistance in ambient air among all the sensors (S0–S6), which is an indication of the maximum depletion of carriers in the catalytic layer. 

The superior response of the multilayer oxide sensors (S4 and S5) compared to the rest of the sensors, was attributed to the optimal content of TiO_2_ uniformly distributed through the volume of the catalytic layers affecting the morphological, electrical and catalytic properties of the sensor. Multilayer structures demonstrated smaller average crystal size after the annealing, higher porosity for 5 and 10% vol. of TiO_2_ and the highest surface roughness across all the sensors.

Based on our studies over a wide temperature range, the pure unmodified SnO_2_ sensor demonstrated relatively poor H_2_S detection capabilities, compared to hybrid (multilayer or bilayer) SnO_2_/TiO_2_ structures. Also, multilayer structures respond better to hydrogen sulfide exposures, than bilayer structures. We relate this phenomenon to the balance between the catalytic activity of the layer and conversion of this catalytic activity into a measurable signal through the charge transfer. The catalytic activity is determined by the surface area of the interactive layer, grain size and structure and by the number of reaction centers (active sites) in the individual grains. The charge transfer that converts catalytic activity into a measurable signal is determined by the oxygen-induced depletion region underneath the oxide surface and by the multiple heterojunctions between the grains. When these factors are balanced, they amplify each other, which was observed in the multilayer oxide structures. In the bilayer structure, the surface depletion was remarkable (even higher than in the multilayer oxides), but the catalytic activity suffered because of the uncontrolled growth of TiO_2_ grains and their agglomeration, which substantially reduced their catalytic activity.

**Figure 7:**
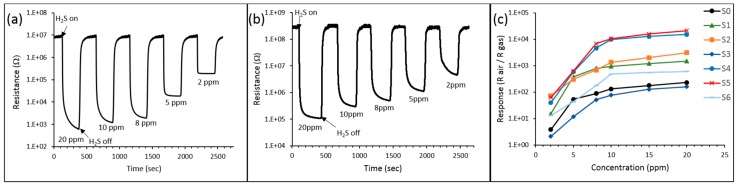
Sensor response of S5 multilayer structure (**a**) and S2 bilayer structure (**b**) to different concentrations of H_2_S (from 2 ppm to 20 ppm). Calibration curves Response vs. Concentration for sensors S0–S6 (**c**).

Response and recovery times were found from the sensor response to 10 ppm of H_2_S under optimal temperature conditions for each sensor (Table 4). Sensors S2 and S5 demonstrated shortest time for the sensor’s response resistance to reach 90% of its steady state value (Figure 7).

**Figure 8:**
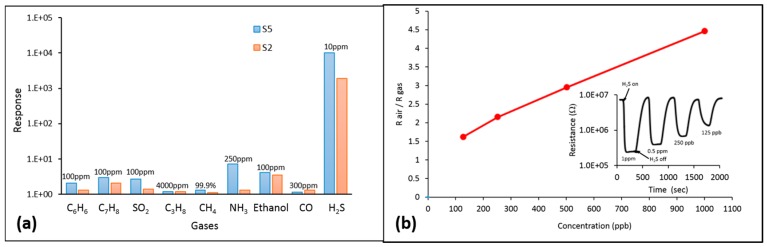
Response amplitudes of sensors S5 (multilayer structure) and S2 (bilayer structure) to various gases (**a**); Response of sensor S5 to sub-ppm concentrations of H_2_S diluted in pure methane (**b**).

Besides excellent sensitivity, quick response and recovery time (Table 4), hybrid sensors also demonstrate superior selectivity to hydrogen sulfide. In our experiments, hybrid sensors were capable of detecting hydrogen sulfide in complex gas mixtures, such as natural gas, which is not typical for metal oxide sensors. The illustration of cross-sensitivity studies on hybrid sensors is shown in the Figure 8. Figure 8a shows the response amplitudes of sensors S5 (multilayer structure) and S2 (bilayer structure) to various gases at different concentrations. Figure 8b shows the response of sensor S5 to sub-ppm concentrations of H_2_S diluted in pure methane. We attribute this remarkable selectivity of hybrid layers to high catalytic activity of SnO_2_/TiO_2_ hybrid structures relative to H_2_S at relatively low temperatures. Maximum sensor response for bilayer and multilayer structures was achieved at 200 °C and 150 °C respectively, which is substantially lower than the optimum activation temperature of pure tin dioxide sensor (300 °C). It is related to a lower activation temperature for oxidation of hybrid catalyst, compared to tin dioxide.

Because of that, the energy of active sites on the surface is not enough to overcome the activation barrier of combustibles, ethanol and carbon dioxide, which provides a natural cut-off for all the catalytic reactions except for the H_2_S decomposition and oxidation.

The major factor that determines chemical sensitivity of a metal oxide sensor is its catalytic activity toward the analyte of interest. Nanoscale titanium dioxide is a very reactive catalyst for the Clauss process and interacts with hydrogen sulfide more efficiently than tin dioxide. Multiple reports show that materials demonstrate the maximum of their catalytic activity in the nanoparticle form, which is related to maximization of the surface area and the number of active sites (reaction centers). In our experiments, both double layer and multilayer-type sensors demonstrated maximum sensitivity at a certain optimum volume percentage of titanium dioxide in the tin dioxide layer. The pure tin dioxide sensor demonstrates moderate sensitivity and no selectivity to hydrogen sulfide. Additionally, a long time of recovery after the exposure is evidence of a relatively low catalytic reaction rate. With an increase of titanium oxide content in the hybrid layer, sensitivity increases dramatically and recovery time drops to a few seconds. Sensors demonstrate the highest sensitivity and the fastest recovery at 10% vol. of titanium oxide. Further increase of titanium oxide content causes the decline of sensor performance, which is associated with agglomeration of titanium nanoparticles into larger grains and reduction of their catalytic activity. Substantial increase in titanium oxide content, overcoming its percolation threshold and formation of a continuous titanium dioxide matrix creates an extremely inert film with no catalytic properties and extremely poor electrical conductivity.

**Figure 9:**
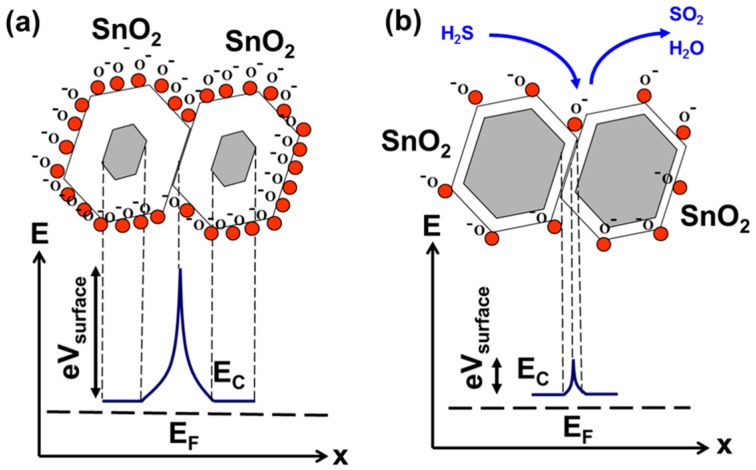
Electronic grain structure of a single-oxide SnO_2_ layer in ambient atmosphere (**a**) and upon exposure to hydrogen sulfide (**b**).

The enhanced sensitivity of a hybrid layer can be better understood considering charge transfer between the grains. The effects of molecular interactions at the surface of metal oxides, and the corresponding changes in their electrical transport properties, has been broadly discussed in literature. A generalized model of sensing is shown in Figure 9. At high temperatures (150 °C–500 °C), intrinsic n-type SnO_2_, once exposed to the ambient air, dissociates and ionizes atmospheric oxygen. The process involves adsorption of *O_2_* molecules, which then trap electrons from the near-surface region of the semiconductor: $O_{2}+e^{-}=O_{2}^{-}$ or $O_{2}+2e^{-}=2O^{-}$. The response to hydrogen sulfide arises from its oxidation and the corresponding stripping of *O*^−^ from the surface of SnO_2_. This, in turn, releases “trapped” electrons back into the bulk. These “released” electrons reduce the width of the depletion layer, concomitant with a reduction in surface band bending, causing an increase in conductance of the SnO_2_. Consistent with the n-type properties of SnO_2_, the Claus process results in peaks in conductance, which corresponds to electron charge transfer back into the bulk.

The two components of the SnO_2_/TiO_2_ hybrid layer are both n-type semiconductors, but they differ significantly in their work function and electron affinity. The work function and electron affinity of TiO_2_ are both around 4.2 eV while the work function of SnO_2_ is around 4.4 eV and its electron affinity is about 0.5 eV larger than that of TiO_2_. The Fermi energy level of TiO_2_ is higher than that of SnO_2_ because of its smaller work function so electron transfer occurs from the conduction band of TiO_2_ to the conduction band of SnO_2_. TiO_2_ and SnO_2_, when crystallized under rutile structure, show very close lattice parameters, favoring the coupling and the heterostructure growth. The formation of the heterojunction and migration of free electrons from the TiO_2_ side to the SnO_2_ side leads to a discontinuity in the conduction band and formation of the energy barrier at the interface [40].

**Figure 10:**
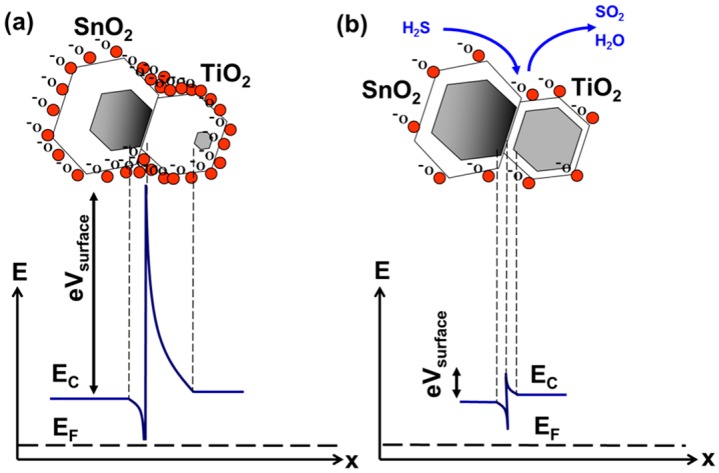
Electronic grain structure of a hybrid SnO_2_/TiO_2_ layer in ambient atmosphere (**a**) and upon exposure to hydrogen sulfide (**b**).

The effects of charge transfer on chemical sensitivity of a hybrid SnO_2_/TiO_2_ layer are shown in Figure 10. In the steady state condition, the formation of an electron-enriched zone at the tin dioxide side of the interface enhances oxygen adsorption in this region [18,41]. Additionally, it leads to a more substantial depletion of titanium dioxide grains and formation of contact potential at the SnO_2_/TiO_2_ boundary. This way the multilayer grain structure of SnO_2_/TiO_2_ layer amplifies trapping of free electrons at the surface, makes the composite layer depletion more extensive and leads to a larger reduction in conductance due to oxygen adsorption, compared to a single-oxide SnO_2_ layer. From our experiments, changes in resistance upon exposure to hydrogen sulfide are much higher for a multilayer heterogeneous system, compared to a single-oxide homogeneous system. This is because in a heterogeneous system more oxygen is available for catalytic surface reactions and more free electrons return back into the bulk during those reactions, than in the homogeneous single-oxide layer. Hence, the changes in resistance upon exposure to hydrogen sulfide are more substantial in the composite layer. Enhanced catalytic activity together with amplified resistance changes due to charge transfer effects make hybrid sensors superior to single-oxide sensors.

**Figure 11:**
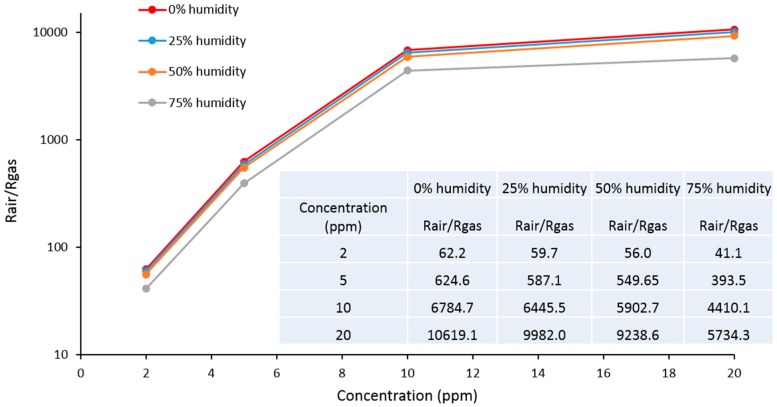
Calibration curves Response vs. Concentration for sensors S5 under different humidity levels.

One of the challenges for metal oxide sensors is the effect of humidity on sensor performance. In our studies, we investigated the operation of sensor S5 under four different humidity levels: 0%, 25%, 50% and 75% (Figure 11). It was found that the humidity level, which is equal or less than 50%, has a minor effect on sensitivity. The total change in sensor response was found to be less than 9% for detection of low H_2_S concentration of 2 ppm and less than 12% for higher concentration of H_2_S of 20 ppm. Further investigation of sensor S5 performance under higher humidity demonstrated that the sensor sensitivity was drastically affected by humidity higher than 75%. The sensor response decreased for both low (2 ppm) and high (20 ppm) concentrations of H_2_S by 33% and 46%, respectively.

Overall sensor performance within humidity range 0%–50% was found to be better than the performance of commercial MOX sensors (Figaro, MQ, etc.) for a wide range of general purpose sensing applications. Additionally, the sensor humidity error correction for different humidity levels could be obtained for more precise measurements. It is important to notice that even at extremely high levels of humidity higher that 75% the high performance of sensors can be achieved by using an additional inline air desiccant.

## 4. Conclusions

On the basis of a hybrid SnO_2_/TiO_2_ structure, a novel metal oxide sensor was developed. Two types of structures were considered: a bilayer structure, where TiO_2_ was deposited on top of SnO_2_, and a multilayer structure, when six layers of TiO_2_ and SnO_2_ were deposited one after another in a sequential manner. Both types of sensors were characterized by multiple techniques in order to relate their geometry, morphology and chemical composition to their catalytic activity. It was found that both types of structures show high catalytic activity at relatively low temperatures, when TiO_2_ content in SnO_2_ is optimized (~10% vol.). Additionally, the optimized hybrid layer demonstrated superior time of response and time of recovery as well as the selectivity to hydrogen sulfide. The multilayer structure demonstrated better operational characteristics than a bilayer structure. The superior performance of the multilayer structure was attributed to the excellent catalytic properties of TiO_2_ nanocrystals, coupled with the SnO_2_ matrix. By optimizing the hybrid layer, charge transfer between the grains amplifies catalytic activity making the layer extremely sensitive. Additionally, the low activation temperature cuts off most of the undesirable catalytic reactions, making the sensor highly selective. The proposed sensor demonstrates performance superior to sensors from the previously published reports and to the commercially available ones.

## Figures and Tables

**Table 1:** Previously reported SnO_2_, SnO_2_ doped and SnO_2_ based sensor characteristics for detection of H_2_S gas.

Material and Structure	Deposition Technique	Concentration (ppm)	Sensitivity (Ra/Rg)	Temperature (°C)	Reference
SnO_2_ porous thick film	Sol gel method	30	30	150	[30]
Sb-SnO_2_ nanoribbons	Thermal evaporation	0.1	10	150	[27]
Ag-SnO_2_ nanocolumns	Glacncing Angle Deposition	5	66	300	[28]
Au-SnO_2_ nanocolumns	Glacncing Angle Deposition	5	111	300	[28]
Ag_2_O-SnO_2_ mesoporous	Nanocasting	0.3	5.7	100	[29]
Fe-SnO_2_ nanoparticles	Pechini method	200	100	25	[32]
TiO_2_/SnO_2_/Fe_2_O_3_ thick film	Hydrothermal method	285	46	40	[31]
CeO_2_–SnO_2_ thin film	Sol gel method	50	23	25	[33]
In_2_O_3_-SnO_2_ thick film	Screen printing	100	1.4 × 10^3^	25	[34]
SnO_2_-ZnO 2D heteronanostructures	Sputtering	0.01	5	100	[35]
CuO-SnO_2_ thin film	Magnetron sputtering	100	1.6 × 10^4^	170	[25]
CuO–SnO_2_ thick film	Screen printing	1	8 × 10^3^	50	[24]
CuO-SnO_2_ bilayer heterostructure	Reactive sputtering	20	7.4 × 10^3^	150	[26]

**Table 2:** Types of structures used in the experiments.

Sensing Material	Composition	Total Thickness	Sample Number
SnO_2_	100%	30 nm	S0
SnO_2_/TiO_2_ bilayer	30 nm + 5 nm	35 nm	S1
SnO_2_/TiO_2_ bilayer	30 nm + 8 nm	38 nm	S2
SnO_2_/TiO_2_ bilayer	30 nm + 20 nm	50 nm	S3
SnO_2_/TiO_2_ multilayer	5% TiO_2_	31.5 nm	S4
SnO_2_/TiO_2_ multilayer	10% TiO_2_	33 nm	S5
SnO_2_/TiO_2_ multilayer	20% TiO_2_	36 nm	S6
TiO_2_	100%	30 nm	S7

**Table 3:** Optimum operational parameters of sensors S0–S6 upon exposure to 10 ppm of H_2_S.

Sample	R Air (Ω)	R Gas (Ω)	R Air/R Gas	Temperature (°C)
S0	1.10 × 10^6^	8.43 × 10^3^	1.31 × 10^2^	225
S1	2.53 × 10^7^	2.64 × 10^4^	9.55 × 10^2^	200
S2	2.50 × 10^8^	1.85 × 10^5^	1.88 × 10^3^	200
S3	8.93 × 10^4^	1.17 × 10^3^	7.60 × 10^1^	250
S4	6.90 × 10^6^	6.99 × 10^2^	9.87 × 10^3^	150
S5	9.39 × 10^6^	8.82 × 10^2^	1.06 × 10^4^	150
S6	3.21 × 10^5^	6.65 × 10^2^	4.83 × 10^2^	150

**Table 4:** Response and recovery time of sensors S0–S6 to 10 ppm of H_2_S.

Sample Number	Response Time T_90_ (s)	Recovery Time T_90_ (s)	Concentration (ppm)	Temperature (°C)
S0	3.7	5.6	10	225
S1	3.5	2.8	10	200
S2	3.3	2.5	10	200
S3	3.7	2.9	10	250
S4	3.0	2.4	10	150
S5	3.2	2.4	10	150
S6	3.9	2.7	10	150

## References

[1] Ou J.Z., Ge W., Carey B., Daeneke T., Rotbart A., Shan W., Wang Y., Fu Z., Chrimes A.F., Wlodarski W. (2015). Physisorption-Based Charge Transfer in Two-Dimensional SnS_2_ for Selective and Reversible NO_2_ Gas Sensing. ACS Nano.

[2] Mei L., Chen Y., Ma J. (2014). Gas Sensing of SnO_2_ Nanocrystals Revisited: Developing Ultra-Sensitive Sensors for Detecting the H_2_S Leakage of Biogas. Sci. Rep..

[3] Verma M.K., Gupta V. (2012). A highly sensitive SnO_2_–CuO multilayered sensor structure for detection of H_2_S gas. Sens. Actuators B Chem..

[4] Wagh M.S., Patil L.A., Seth T., Amalnerkar D.P. (2004). Surface cupricated SnO_2_–ZnO thick films as a H_2_S gas sensor. Mater. Chem. Phys..

[5] Xi L., Qian D., Tang X., Chen C. (2008). High surface area SnO_2_ nanoparticles: Synthesis and gas sensing properties. Mater. Chem. Phys..

[6] Hwang I.-S., Choi J.-K., Kim S.-J., Dong K.-Y., Kwon J.-H., Ju B.-K., Lee J.-H. (2009). Enhanced H_2_S sensing characteristics of SnO_2_ nanowires functionalized with CuO. Sens. Actuators B Chem..

[7] Chen J., Wang K., Hartman L., Zhou W. (2008). H_2_S Detection by Vertically Aligned CuO Nanowire Array Sensors. J. Phys. Chem. C.

[8] Kaneti Y.V., Yueb J., Moriceauc J., Chena C., Liud M., Yuana Y., Jiangd X., Yua A. (2015). Experimental and theoretical studies on noble metal decorated tin oxide flower-like nanorods with high ethanol sensing performance. Sens. Actuators B Chem..

[9] Tang Y., Su B., Liu M., Feng Y., Jiang X., Jiang L., Yu A. (2016). Superwettability Strategy: 1D Assembly of Binary Nanoparticles as Gas Sensors. Small.

[10] Yue J., Jiang X., Yu A. (2013). Adsorption of the OH Group on SnO_2_ (110) Oxygen Bridges: A Molecular Dynamics and Density Functional Theory Study. J. Phys. Chem. C.

[11] Huang J., Wan Q. (2009). Gas Sensors Based on Semiconducting Metal Oxide One-Dimensional Nanostructures. Sensors.

[12] Das S., Jayaraman V. (2014). SnO_2_: A comprehensive review on structures and gas sensors. Prog. Mater. Sci..

[13] Naik A., Parkin I., Binions R. (2016). Gas Sensing Studies of an n-n Hetero-Junction Array Based on SnO_2_ and ZnO Composites. Chemosensors.

[14] Yamazoe N., Tamaki J., Miura N. (1996). Role of hetero-junctions in oxide semiconductor gas sensors. Mater. Sci. Eng. B.

[15] Costello L., Ewen R.J., Ratcliffe N.M., Sivanand P. (2003). Thick film organic vapour sensors based on binary mixtures of metal oxides. Sens. Actuators B Chem..

[16] Yamazoe N., Shimanoe K. (2013). Proposal of contact potential promoted oxide semiconductor gas sensor. Sens. Actuators B Chem..

[17] Halek P., Teterycz H., Halek G., Suchorska P., Wiśniewski K. Sensing performance of heterojunction gas sensors based on SnO_2_, WO_3_ and ZnO metal oxides. Proceedings of the IMCS 2012—The 14th International Meeting on Chemical Sensors.

[18] Suchorska-Woźniak P., Rac O., Fiedot M., Teterycz H. (2014). Analysis of SnO_2_|WO_3_ Heterocontact Properties during the Detection of Hydrogen Sulphide. Sensors.

[19] Wager J.F. (2008). Transparent electronics: Schottky barrier and heterojunction considerations. Thin Solid Films.

[20] Jiao Z., Wang S., Bian L., Liu J. (2000). Stability of SnO_2_/Fe_2_O_3_ multilayer thin film gas sensor. Mater. Res..

[21] Guo W., Mei L., Wenc J., Ma J. (2016). High-response H_2_S sensor based on ZnO/SnO_2_ heterogeneous nanospheres. RSC Adv..

[22] Huang W.-F., Chen H.-T., Lin M.C. (2009). Density Functional Theory Study of the Adsorption and Reaction of H_2_S on TiO_2_ Rutile (110) and Anatase (101) Surfaces. J. Phys. Chem. C.

[23] Chun S.W., Jang J.Y., Park D.W., Woo H.C., Chung J.S. (1998). Selective oxidation of H_2_S to elemental sulfur over TiO_2_/SiO_2_ catalysts. Appl. Catal. B.

[24] Garcia A., Yan N., Vincent A., Singh A., Hill J.M., Chuang K.T., Luo J.-L. (2015). Highly cost-effective and sulfur/coking resistant VO*_x_*-grafted TiO_2_ nanoparticles as an efficient anode catalyst for direct conversion of dry sour methane in solid oxide fuel cells. J. Mater. Chem. A.

[25] Zhang X., Tang Y., Qu S., Da J., Hao Z. (2015). H_2_S-Selective Catalytic Oxidation: Catalysts and Processes. ACS Catal..

[26] Radecka M., Zakrzewska K., Rękas M. (1998). SnO_2_–TiO_2_ solid solutions for gas sensors. Sens. Actuators B Chem..

[27] Patil L.A., Patil D.R. (2006). Heterocontact type CuO-modified SnO_2_ sensor for the detection of a ppm level H_2_S gas at room temperature. Sens. Actuators B Chem..

[28] Vasiliev R.B., Rumyantsev M.N., Yakovlev N.V., Gaskov A.M. (1998). CuO/SnO_2_ thin film heterostructures as chemical sensors to H_2_S. Sens. Actuators B Chem..

[29] Chowdhuri A., Singh S.K., Sreenivas K., Gupta V. (2010). Contribution of adsorbed oxygen and interfacial space charge for enhanced response of SnO_2_ sensors having CuO catalyst for H_2_S gas. Sens. Actuators B Chem..

[30] Ma J., Liu Y., Zhang H., Ai P., Gong N., Wu Y., Yu D. (2015). Room temperature ppb level H_2_S detection of a single Sb-doped SnO_2_ nanoribbon device. Sens. Actuators B Chem..

[31] Yoo K.S., Han S.D., Moon H.G., Yoon S.-J., Kang C.-Y. (2015). Highly Sensitive H_2_S Sensor Based on the Metal-Catalyzed SnO_2_ Nanocolumns Fabricated by Glancing Angle Deposition. Sensors.

[32] Yang T., Yang Q., Xiao Y., Sun P., Wang Z., Gao Y., Ma J., Sun Y., Lu G. (2016). A pulse-driven sensor based on ordered mesoporous Ag_2_O/SnO_2_ with improved H_2_S-sensing performance. Sens. Actuators B Chem..

[33] Liua H., Gonga S.P., Hua Y.X., Liua J.Q., Zhoua D.X. (2009). Properties and mechanism study of SnO_2_ nanocrystals for H_2_S thick-film sensors. Sens. Actuators B Chem..

[34] Pedhekar R.B., Raghuwanshi F.C., Kapse V.D., Raisoni G.H. (2015). Low Temperature H_2_S Gas Sensor Based on Fe_2_O_3_ Modified ZnO-TiO_2_ Thick Film. Int. J. Mater. Sci. Eng..

[35] Vaishampayan M.V., Deshmukh R.G., Walke P., Mulla I.S. (2008). Fe-doped SnO_2_ nanomaterial: A low temperature hydrogen sulfide gas sensor. Mater. Chem. Phys..

[36] Fang G., Liu Z., Liu C., Yao K. (2000). Room temperature H_2_S sensing properties and mechanism of CeO_2_–SnO_2_ sol–gel thin films. Sens. Actuators B Chem..

[37] Liu H., Wu S., Gong S., Zhao J., Liu J., Zhou D. (2011). Nanocrystalline In_2_O_3_–SnO_2_ thick films for low-temperature hydrogen sulfide detection. Ceram. Int..

[38] Fu D., Zhu C., Zhang X., Lia C., Chen Y. (2016). Two-dimensional net-like SnO_2_/ZnO heteronanostructures for high-performance H_2_S gas sensor. J. Mater. Chem. A.

[39] Tang H., Prasad K., Sanjinès R., Schmid P.E., Lévy F. (1994). Electrical and optical properties of TiO_2_ anatase thin films. J. Appl. Phys..

[40] Floriano E.A., Scalvi L.V.A., Saeki M.J., Sambrano J.R. (2014). Preparation of TiO_2_/SnO_2_ Thin Films by Sol−Gel Method and Periodic B3LYP Simulations. J. Phys. Chem. A.

[41] Trakhtenberg L.I., Gerasimov G.N., Gromov V.F., Belysheva T.V., Ilegbusi O.J. (2012). Gas Semiconducting Sensors Based on Metal Oxide Nanocomposites. J. Mater. Sci. Res..

