# Addendum: Larin, A.; Womble, P.C.; Dobrokhotov, V. Hybrid SnO_2_/TiO_2_ Nanocomposites for Selective Detection of Ultra-Low Hydrogen Sulfide Concentrations in Complex Backgrounds. *Sensors* 2016, 16, 1373.

**DOI:** 10.3390/s17040768

**Published:** 2017-04-05

**Authors:** Alexander Larin, Phillip C. Womble, Vladimir Dobrokhotov

**Affiliations:** 1 VAON LLC, KY, USA, 2200 Lapsley Lane, Bowling Green, KY 42103, USA; alexander.larin@bgfky.com (A.L.); phillip.womble@bgfky.com (P.C.W.); 2 Department of Physics and Astronomy, University of Louisville, Louisville, KY 40292, USA; 3 Applied Physics Institute, Western Kentucky University, Bowling Green, KY 42101, USA

The authors wish to make the following correction to their paper [1]:

The first author of the paper, Alexander Larin, is a Research Engineer at Vaon LLC and also a graduate student at the University of Louisville. Therefore, one more affiliation is added to this author: “Department of Physics and Astronomy, University of Louisville, Louisville, KY 40292, USA”.

The authors would like to apologize for any inconvenience caused to the readers by this change. The manuscript will be updated and the original will remain online on the article webpage.

## Supplementary Materials

Supplementary File 1
