# Supplementary material for: Addendum: Larin, A.; Womble, P.C.; Dobrokhotov, V. Hybrid SnO2/TiO2 Nanocomposites for Selective Detection of Ultra-Low Hydrogen Sulfide Concentrations in Complex Backgrounds. Sensors 2016, 16, 1373
Source: Sensors (Basel). 2017 Apr 5;17(4):768. doi: 10.3390/s17040768 (PMC5422041; doi:10.3390/s17040768)
Supplement: Supplementary File 1 [file sensors-17-00768-s001.zip › sensors-16-01373.pdf]

sensors

MDPI

Article

Hybrid SnO_2/TiO_2 Nanocomposites for Selective Detection of Ultra-Low Hydrogen Sulfide Concentrations in Complex Backgrounds

Alexander Larin [1,2], Phillip C. Womble [1] and Vladimir Dobrokhotov [3,*]

[1] VAON LLC, KY, USA, Bowling Green, KY 42101, USA; alexander.larin@bgfky.com (A.L.); phillip.womble@bgfky.com (P.C.W.)
[2] Department of Physics and Astronomy, University of Louisville, Louisville, KY 40292, USA
[3] Applied Physics Institute, Western Kentucky University, Bowling Green, KY 42101, USA
[*] Correspondence: vladimir.dobrokhotov@wku.edu; Tel.: +1-270-745-6201

Academic Editor: W. Rudolf Seitz
Received: 16 July 2016; Accepted: 19 August 2016; Published: 27 August 2016

Abstract: In this paper, we present a chemiresistive metal oxide (MOX) sensor for detection of hydrogen sulfide. Compared to the previous reports, the overall sensor performance was improved in multiple characteristics, including: sensitivity, selectivity, stability, activation time, response time, recovery time, and activation temperature. The superior sensor performance was attributed to the utilization of hybrid SnO_2/TiO_2 oxides as interactive catalytic layers deposited using a magnetron radio frequency (RF) sputtering technique. The unique advantage of the RF sputtering for sensor fabrication is the ability to create ultra-thin films with precise control of geometry, morphology and chemical composition of the product of synthesis. Chemiresistive films down to several nanometers can be fabricated as sensing elements. The RF sputtering technique was found to be very robust for bilayer and multilayer oxide structure fabrication. The geometry, morphology, chemical composition and electronic structure of interactive layers were evaluated in relation to their gas sensing performance, using scanning electron microscopy (SEM), X-ray diffraction technique (XRD), atomic force microscopy (AFM), Energy Dispersive X-ray Spectroscopy (EDAX), UV visible spectroscopy, and Kelvin probe measurements. A sensor based on multilayer SnO_2/TiO_2 catalytic layer with 10% vol. content of TiO_2 demonstrated the best gas sensing performance in all characteristics. Based on the pattern relating material's characteristics to gas sensing performance, the optimization strategy for hydrogen sulfide sensor fabrication was suggested.

Keywords: sensor; micro-electromechanical systems (MEMS); hydrogen sulfide; nanocomposites

---

1. Introduction

Hydrogen sulfide is a hazardous gas with strong odor. Lower concentrations of it can cause nausea, vomiting and eye irritation. Higher concentrations of hydrogen sulfide can lead to shock, convulsions, inability to breathe, and coma. It is extremely important to detect hydrogen sulfide leaks at early stages of exposure in order to prevent poisoning. Detection of hydrogen sulfide plays an important role in oil and gas exploration and production.

Some of the major H_2S monitoring technologies include electrochemical, infrared and metal oxide sensors. Recently developed physisorptive gas sensor technology is based on charge transfer in two-dimensional tin disulfide at low operating temperatures [1]. Gas sensors based on electrochemical and MOX gas sensing technologies hold the largest market share of around 21% and 20%, respectively. Better efficiency, fast response time, and cost effectiveness are some important factors that led to the growth of gas sensors based on these technologies. Some of the major players engaged in gas sensors

manufacturing include City Technology, Alphasense, Membrapor, Dynament, Figaro Engineering, and others. At the same time, state-of-the-art electrochemical and infrared sensor technologies already met their technical limitations. In the last decade there was a very limited change in design, physical dimensions, power consumption or the cost of these sensors. Their performance in terms of sensitivity, selectivity, stability or a lifetime have not been significantly improved either.

Metal oxide (MOX) sensors are widely used for hydrogen sulfide monitoring [2–7]. For more than two decades now, small and simple gas sensors have been commercially available. Typically, they are manufactured by the sol-gel method, in which metal oxide layers are deposited in the form of viscous paste and then baked in the inert environment, creating thick films. Hydrogen sulfide MOX sensors from Figaro (TGS sensors) and Henan Hanwei Electronics Co., Ltd. (Henan, China) (MQ sensors) are manufactured using this method. Most of the commercial metal oxide sensors utilize purely macroscopic solutions and their dimensions are comparable with infrared and electrochemical sensors. At the same time, the novel innovative nanotechnology and MEMS-based solutions are currently available primarily in the area of metal oxide sensors.

Recently, a great deal of effort has been focused on utilizing nanostructures for sensor applications. Significant advantages of nanomaterials as sensitive elements arise from their extremely high surface-to-volume ratio, which makes their electromechanical and thermal properties strongly dependent on surface phenomena. The condition of the surface of the nanostructure is determined by the parameters of the surrounding medium, which allows one to establish a direct correspondence between the properties of the nanostructure and the parameters of the medium in which this nanostructure is immersed. This basic principle drives the sensing mechanisms of most nanomaterials. Kaneti et al. [8] conducted experimental and theoretical studies of ethanol sensing by Au and Pd decorated tin oxide flower-like nanorods. It was found that deposition of Au and Pd nanoparticles on the surface of the SnO_2 nanorods is advantageous in: enhancing the sensitivity towards ethanol (9–15 times); reducing the response/recovery time (by 15–40 s), and significantly decreasing the optimum operating temperature (from 250 to 175 °C). Tang et al. [9] studied 1-D assembly of binary nanoparticles as gas sensors. Binary 1-D nanowires consisting of both SnO_2 nanoparticles and Au nanorods, fabricated through a "substrate–particle solution template" assembling method, demonstrated highly enhanced gas sensitivity toward acetone under ambient conditions. Yue et al. [10] conducted a density functional theory study of adsorption of the OH Group on SnO_2 Oxygen Bridges. The authors analyzed factors influencing gas sensing performance of SnO_2 sensors. The adsorption of water, oxygen, ethanol, and acetic acid onto the SnO_2 (110) surface was studied using molecular dynamics (MD) simulations.

Various nanomaterials demonstrate high sensitivity toward hydrogen sulfide: nanocrystals, nanofibers, nanowires, nanosprings, nanoribbons, etc. [2,6,7,11]. However, their thermal and electrical stability over an extended period of time in complex backgrounds is lacking. Additionally, reproducibility of nanomaterials-based sensors is limited, which complicates the scalable manufacturing techniques and commercial mass production of this type of sensors. In contrast to nanomaterials, metal oxide thin films are very stable and reproducible, but limited in sensitivity. The current effort in sensor technology is concentrated on development of devices combining the properties of nanomaterials and thin films, which are very sensitive and selective and at the same time stable and reproducible.

There is an interest toward developing a high-quality MOX sensor for detection of H_2S. Tin oxide (SnO_2) is one of the most common chemiresistive materials due to its high catalytic activity toward a broad range of analytes [2,5,12]. It has been found that certain semiconductor additives improve the performance of tin oxide layers in terms of sensitivity and selectivity, compared to bare tin oxide. It was reported that grain boundaries between different oxides have higher catalytic activity, compared to single-oxide grain boundaries [13–17]. Recently, SnO_2-based metal oxide structures with n-p and n-n heterojunction have attracted a great deal of interest as gas sensing materials [2,13–19].

The concept of electron transport through the grain boundary from material with lower work function to material with higher work function with the formation of contact potential in the

equilibrium state has been used to describe the electron distribution at the heterojunction [20]. The role of heterojunctions in molecular adsorption and catalytic reactions was considered by Yamazoe et al. [14]. In this work, the authors proposed how the contact potential at the interface of different oxides affects the electron transport through the contact. It was also suggested that the heterojunction resistance at the interface of two different oxides is more sensitive to the local gas environment than the contact resistance at the interface of two grains of the same nature. This means that the content of the multilayer oxide structure can be optimized in order to achieve maximum sensing performance.

Enhanced detection of H_2S has been reported by using different heterojunction structures SnO_2/CuO, SnO_2/WO_3 and SnO_2/ZnO [3,4,18,21]. The sensitivity of multilayer oxides was found to be close to the sensitivity of novel nanomaterials, but at the same time the thin film sensors demonstrated high stability and repeatability. Among other advantages of utilization of multilayer oxides in chemiresistors are low activation temperature, short activation time, short time of response and short time of recovery.

One of the challenges for hydrogen sulfide detection by metal oxides is sulfur poisoning. Many sensors lose their catalytic activity over time. TiO_2 catalyst and catalyst support is known for its high sulfur resistance [22–25]. It is one of the catalysts that are widely used in industry for H_2S decomposition and oxidation, also known as the Claus process. Even though TiO_2 is one of the most interactive with H_2S catalysts, it is a challenge to use it as a sensing element due to its very low electrical conductivity. At the same time, excellent thermal, mechanical, and catalytic properties makes it a good candidate for doping material [26–31]. The results obtained by various research groups on detection of H_2S by using metal oxides are summarized in Table 1.

Table 1. Previously reported SnO_2, SnO_2 doped and SnO_2 based sensor characteristics for detection of H_2S gas.

Material and Structure	Deposition Technique	Concentration (ppm)	Sensitivity (Ra/Rg)	Temperature (°C)	Reference
SnO_2 porous thick film	Sol gel method	30	30	150	[30]
Sb-SnO_2 nanoribbons	Thermal evaporation	0.1	10	150	[27]
Ag-SnO_2 nanocolumns	Glacncing Angle Deposition	5	66	300	[28]
Au-SnO_2 nanocolumns	Glacncing Angle Deposition	5	111	300	[28]
Ag_2O-SnO_2 mesoporous	Nanocasting	0.3	5.7	100	[29]
Fe-SnO_2 nanoparticles	Pechini method	200	100	25	[32]
$TiO_2/SnO_2/Fe_2O_3$ thick film	Hydrothermal method	285	46	40	[31]
CeO_2–SnO_2 thin film	Sol gel method	50	23	25	[33]
In_2O_3-SnO_2 thick film	Screen printing	100	1.4×10^3	25	[34]
SnO_2-ZnO 2D heteronanostructures	Sputtering	0.01	5	100	[35]
CuO-SnO_2 thin film	Magnetron sputtering	100	1.6×10^4	170	[25]
CuO–SnO_2 thick film	Screen printing	1	8×10^3	50	[24]
CuO-SnO_2 bilayer heterostructure	Reactive sputtering	20	7.4×10^3	150	[26]

There are several well-known deposition methods for sensing element fabrication: sol-gel method, screen printing, thermal evaporation, nanocasting etc. [32–38] RF magnetron sputtering technique is a well-known method for thin film fabrication. The unique advantage of the RF sputtering for sensor fabrication is the ability to create ultra-thin films with precise control over geometry, morphology and chemical composition of the product of synthesis. Chemiresistive films down to several nanometers can be fabricated as sensing elements. The RF sputtering technique was found to be very robust for bilayer and multilayer oxide structure fabrication. We will call a bilayer structure a thin film obtained

by a consecutive deposition of two oxides. We will call a multilayer structure a thin film obtained by multiple consecutive depositions of two oxides.

In the present work, novel multilayer and bilayer SnO_2/TiO_2 structures were evaluated for H_2S detection. The effect of SnO_2/TiO_2 n-n heterojunction on sensing performance was studied. Thickness, morphology, and activation temperature of the sensing element were optimized for maximum sensor response. Sensitivity, selectivity, stability, response and recovery time as well as the resistance to sulfur poisoning superior to the previously published data and commercial analogs were achieved as a result of this study.

2. Experimental Section

2.1. Fabrication and Characterization of a Suspended Microheater Platform

A MEMS-based suspended membrane with a cross-shaped heating element and interdigitated sensor's electrodes was fabricated for precise temperature control over the interactive metal oxide layer (Figure 1).

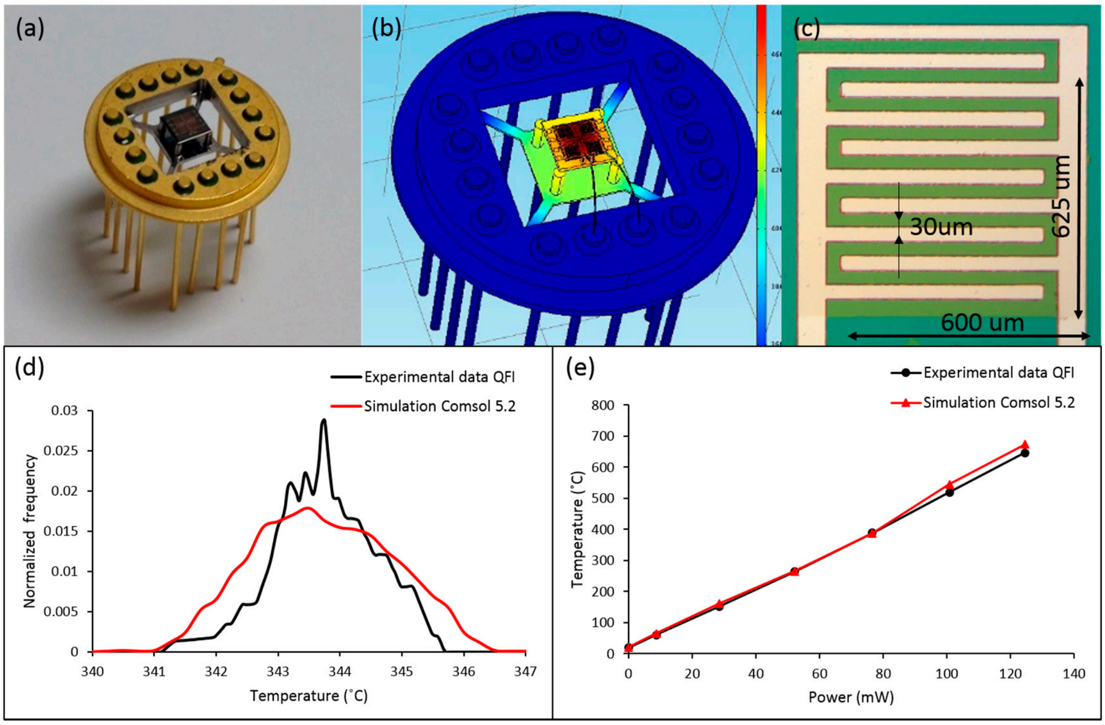

Figure 1. (**a**) Simulated temperature distribution over the microplatform attached to a TO package; (**b**) optical images of the sensor platform attached to a modified TO package; (**c**) a single sensing element area; (**d**) simulated and experimental temperature distribution across the sensing area under 70 mW heating power; (**e**) simulated and experimental data of the sensor average temperature over different power dissipation 8–120 mW.

The microplatform contains four identical square sensor elements in the corners and a cross-shaped heater in the center of the platform. The hot plate was cleanroom fabricated by a multi-step procedure: photolithography, sputtering deposition, liftoff, back side photolithography, reactive ion etching, deep reactive ion etching, and dicing. The heating element and interdigitated sensor's electrodes were made out of 300 nm platinum deposited by magnetron sputtering (PVD 75 Lesker). A 5 nm buffer layer of titanium was deposited prior to platinum deposition for better adhesion. The membrane structure was fabricated by Deep Reactive Ion Etching (DRIE). The membrane thickness 50 µm was verified by surface profilometer KLA-Tencor Alpha-Step IQ. The surface temperature on

the suspended membrane was first simulated using Comsol 5.2 software (COMSOL Inc., Stockholm, Sweden) and then compared with the experimental data from Quantum Focus Instruments (QFI) thermal imaging system, which is able to capture thermal images of the platform featuring 0.1 °C temperature and 5 μm spatial resolutions. The microplatform temperature profiles were obtained for different values of power dissipation across the heating element. It was also found that the temperature gradient along the sensing element area does not exceed 5 °C when the average surface temperature is above 350 °C. The microplatform was developed for four identical sensors in order to obtain small statistics over sensor response and stability characteristics. Although in the present study the sensitive elements on the platform are identical, the future intent is to expand the scope of this research by depositing different interactive layers on the same platform.

2.2. Oxides Deposition and Characterization

Thin films of TiO_2, SnO_2, SnO_2/TiO_2 multilayer structure and SnO_2/TiO_2 bilayer structure were deposited by RF magnetron sputtering using SnO_2 and TiO_2 three inch targets with purity of 99.99% and 99.998%, respectively. The sample rotation speed during the deposition was set up to 30 rpm for equal thickness distribution across the sample area. The deposition was conducted at room temperature and no special bias voltage was applied to the wafer. The schematics of a single-layer, a bilayer and a multilayer are shown in Figure 2a–c, respectively. The single-oxide samples (SnO_2, TiO_2) were sputtered under 12 mTorr of argon (Ar) pressure and RF power 200 W. The bilayer samples of TiO_2/SnO_2 were fabricated during the two step process: main layer deposition (SnO_2) and surface doping layer deposition (TiO_2). The multilayer TiO_2/SnO_2 structures were prepared by multiple consequent sputtering of two metal oxides. Different volume contents of TiO_2 in SnO_2 were obtained by varying power of the TiO_2 source during its deposition phase. The multilayer structure was constructed out of a total of 6 layers: 3 layers of SnO_2 and 3 layers of TiO_2. After the deposition, the total thickness of each sample was verified by surface contact profilometer (KLA-Tencor 500 Alpha-Step IQ, (TENCOR Instruments, Mountain View, CA, USA). During the sample preparation eight different sample structures were prepared (Table 2). After the deposition, all the samples were annealed in a tube furnace (MKS OTF 1200x, MTI Corporaion, Richmond, CA, USA) under 500 °C for 48 h in ultra-zero grade air (UZ300 Airgas 100 sccm).

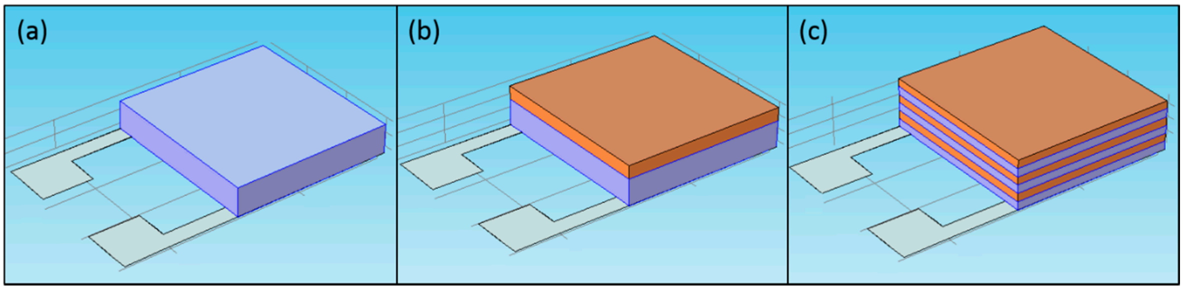

Figure 2. The schematics of a single-layer (**a**); a bilayer (**b**); and a multilayer (**c**).

Table 2. Types of structures used in the experiments.

Sensing Material	Composition	Total Thickness	Sample Number
SnO_2	100%	30 nm	S0
SnO_2/TiO_2 bilayer	30 nm + 5 nm	35 nm	S1
SnO_2/TiO_2 bilayer	30 nm + 8 nm	38 nm	S2
SnO_2/TiO_2 bilayer	30 nm + 20 nm	50 nm	S3
SnO_2/TiO_2 multilayer	5% TiO_2	31.5 nm	S4
SnO_2/TiO_2 multilayer	10% TiO_2	33 nm	S5
SnO_2/TiO_2 multilayer	20% TiO_2	36 nm	S6
TiO_2	100%	30 nm	S7

The crystal structure of samples was evaluated by X-ray diffraction method (XRD). The XRD spectrum of samples was collected by a Thermo ARL (model XTRA, Thermo Fisher Scientific Waltham, MA, USA) X-ray diffraction machine (Cu Kα radiation wavelength was 0.15056 nm). A scanning electron microscope (SEM Zeiss Supra 35, Carl Zeiss AG, Oberkochen, Germany) was utilized to study the surface microstructure of the samples. The chemical composition of the samples was obtained by Energy Dispersive X-ray Spectroscopy (EDAX) analysis. The band gap and work function of tin oxide and titanium oxide were evaluated by UV visible spectrometer AvaSpec-ULS2048L-EVO (Avantes, Louisville, CO, USA) and Kelvin probe measurement, respectively.

2.3. Gas Delivery System and Data Acquisition

The schematic of gas delivery and data acquisition system is shown in Figure 3. Sensor response characterization was conducted in a small (1 cm^3) environmental chamber. The total flow rate through the chamber was fixed at 100 sccm during the experiment. All the data was collected under atmospheric pressure. The sensor resistance was measured with the Keithley 3706 system (Keithley Instruments, Cleveland, OH, USA) switch/multimeter connected to a PC through Labview interface. The resistance of the sensor was measured with a sample rate of 10 Hz. A system of mass flow controllers (Omega GMA 2709 and MKS 1478A, OMEGA Engineering, INC., Stanford, CT, USA) interfaced with Labview through PCI NI 6251 and BNC21110 was utilized to produce specific concentrations of the target gas. The gas delivery sistem was able to produce different concentrations of hydrogen sulfide in a range from 125 ppb to 200 ppm. The exposure time was chosen to be 4 min. During this time, the sensor signal was able to reach the saturation point for a particular concentration. After exposure, the chamber was flashed with 100 sccm of clean dry sinthetic air until the sensor signal returned to its original baseline. The sensor response was defined as $S = R_{Air}/R_{Gas}$, where R_{Air} is the sensor resistance in clean dry air and R_{Gas} is the sensor resistance upon exposure to hydrogen sulfide.

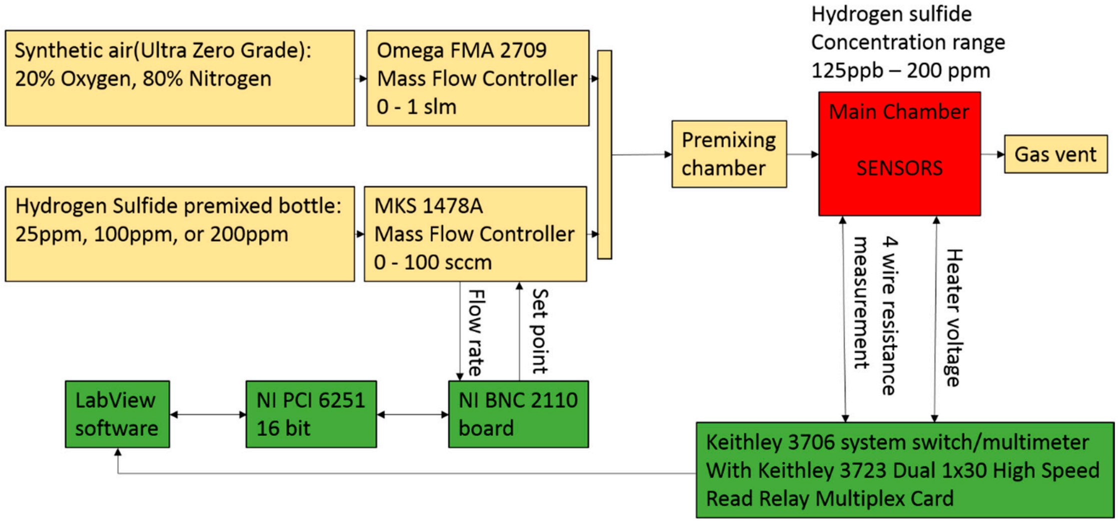

Figure 3. Gas delivery and data collection system.

3. Results and Discussion

After the deposition, samples were annealed at 500 °C in the tubular furnace for 48 h. The choice of platinum as a material for electrodes and heater fabrication is crucial for preservation of the device stability under annealing. It was chosen for its exceptional chemical resistance in combination with high mechanical, electrical and thermal stability over a wide range of temperatures. The melting point of platinum (1768.3 °C) is much higher than the average annealing temperature (300–900 °C) for most of the metal oxides. Platinum electrodes can also be used for extreme annealing temperatures 1000–1200 °C, if necessary.

After the annealing, a nanocrystalline structure of oxides was observed by X-ray diffraction (XRD). The XRD patterns were recorded at a scanning rate of 1.2 times per second and a scanning step size of 0.02°. The XRD patterns were received from larger (1 × 1 inch) glass samples. These samples were prepared simultaneously with sensor fabrication. The simultaneous and uniform coating of several samples in a single run is one of the advantages of the reactive ion sputtering technique. A continuous rotation of the sample holder stage during the deposition process assures a uniform coating over the entire group of samples. The scanning range for all the samples was from 20° to 60°. Figure 4 shows the XRD pattern of all the samples (S0–S7). The XRD spectrum of pure SnO_2 (S0) showed strong diffraction peaks at 2θ = 26.92°, 34.22°, 38.22°, and 52.17° corresponding to (110), (101), (200) and (211) crystal faces of rutile structure of SnO_2 [20]. The anatase structure of TiO_2 was identified by the major diffraction peak at 2θ = 25.43° [39]. The XRD analysis of the bilayer samples S1, S2 and S3 showed diffraction peaks similar to SnO_2 crystal structure and additional peak at 2θ = 25.4° corresponding to (101) crystal faces of anatase structure of TiO_2 was detected for samples S2 and S3. The XRD analysis of the composite SnO_2/TiO_2 structure (S4–S6) revealed three major peaks similar to SnO_2 (S0). The position of the major diffraction peak of multilayer oxides shifts slightly from 2θ = 26.78° (S6) to 2θ = 26.87° (S5) and 2θ = 26.91° (S4) with decreasing % vol. of TiO_2. In addition, the average crystal size of all the samples based on the major diffraction peak was calculated by using the Scherrer formula (Equation (1)):

$$D = \frac{K\lambda}{\beta \cos(\theta)} \tag{1}$$

where D is the average size of nanocrystals, λ is the X-ray wavelength (1.5056 nm), β is the line broadening at half the maximum peak intensity (FWHM), K = 0.9 is a dimensionless shape factor and θ is the major diffraction peak position. The average size of SnO_2 (S0) nanocrystals after the annealing process was found to be d = 7.87 nm. The characteristic size of nanocrystals for a multilayer SnO_2/TiO_2 structure was found to be smaller, compared to pure SnO_2: d = 4.87 nm (S4), d = 4.54 nm (S5) and d = 4.09 nm (S6). The crystal size of the TiO_2 (S7) was calculated to be 4.21 nm. The smaller grain size of the composite oxides (S4–S6) could be an advantage for gas sensing properties. During the XRD analysis, samples S4–S6 showed no specific peaks correlated to TiO_2 crystal structure. However, a noticeable asymmetry, as well as a slight shift in the major peak of the multilayer structure in Figure 4c, may be attributed to the overlap of TiO_2 and SnO_2 peaks, caused by the small TiO_2 nanocrystals present in the layer.

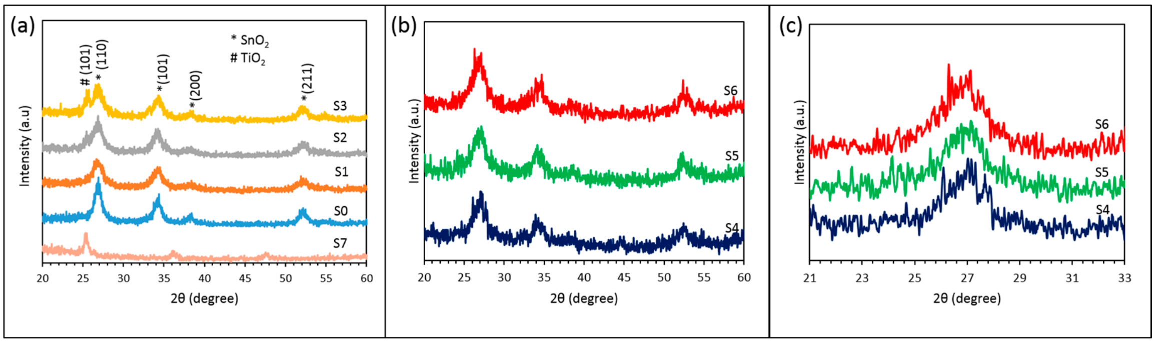

Figure 4. XRD spectroscopy of the (**a**) samples S0–S3 and S7; (**b**) samples S4–S6; and (**c**) zoom in on the major peaks of the samples S4–S6.

The morphology of samples S0–S7 was also studied by SEM (Zeiss Supra 35), as shown in Figure 5. All the samples demonstrated rough and porous polycrystalline structure with short neck-like interconnections between the grains. It can be seen that the porosity of the samples S0–S3 was gradually decreasing with the increasing content of TiO_2. The SEM software analysis was used to determine the

average grain size of the samples S0–S7. The grain size of the pure SnO_2 (S0) and SnO_2/TiO_2 (S1–S3) bilayer structures from SEM analysis were found to be in a range of 10–15 nm.

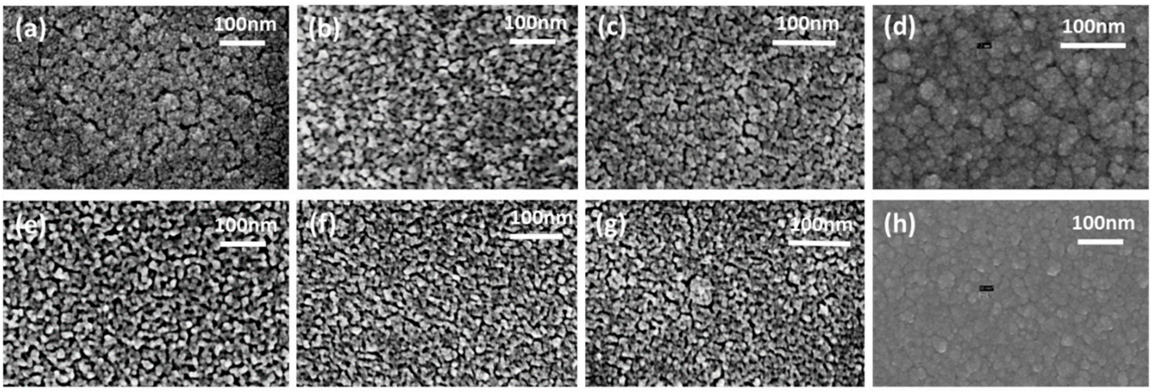

Figure 5. SEM images of the crystalline structure of samples S0–S7: (**a**) SnO_2 (S0); (**b**) SnO_2/TiO_2 bilayer structure 30 nm + 5 nm (S1); (**c**) SnO_2/TiO_2 bilayer structure 30 nm + 10 nm (S2); (**d**) SnO_2/TiO_2 bilayer structure 30 nm + 20 nm (S3); (**e**) SnO_2/TiO_2 multilayer 5% of TiO_2 (S4); (**f**) SnO_2/TiO_2 multilayer 20% of TiO_2 (S5); (**g**) SnO_2/TiO_2 multilayer 50% of TiO_2 (S6); (**h**) TiO_2 (S7).

The SnO_2/TiO_2 (S4–S6) multilayer structure also showed a slight decrease in porosity with increasing of TiO_2 content from 5% vol. to 20% vol. The grain size of the multilayer SnO_2/TiO_2 structures (S4–S6) was in the range of 5–10 nm. It was found that sample S4 has more uniform grain size distribution and higher porosity in comparison to the rest of the samples. The combination of small grains with high porosity of samples S4, S5 and S6 creates favorable conditions for catalytic reactions thanks to the large surface area and high number of active sites.

Sensor Performance Characteristics

The sensors' (S0–S7) performance characteristics were first investigated over a wide temperature range of 100–350 °C for exposures to 10 ppm of H_2S in synthetic air (Figure 6). The optimized temperature conditions for H_2S detection were found for each sensor S0–S6 (Table 3). The pure TiO_2 (S7) sample did not show any noticeable response to 10 ppm of H_2S over the temperature range 100–350 °C. The bilayer SnO_2/TiO_2 structure (S2) demonstrated a much higher response of 1.88×10^3 to 10 ppm to H_2S gas at lower temperature of 200 °C compared to pure SnO_2 (S0) sensitivity of 1.31×10^2 at 225 °C. The highest sensor response to 10 ppm of H_2S of 1.06×10^4 was observed for SnO_2/TiO_2 (S5) composite structure at an even lower temperature of 150 °C. It was demonstrated that SnO_2/TiO_2 multilayer material has superior sensitivity toward H_2S at lower temperatures. The sensor performance characteristics of the SnO_2 based sensor with 10% vol. of TiO_2 (S5) was found to be more efficient compared to the other results from previous reports (Table 1).

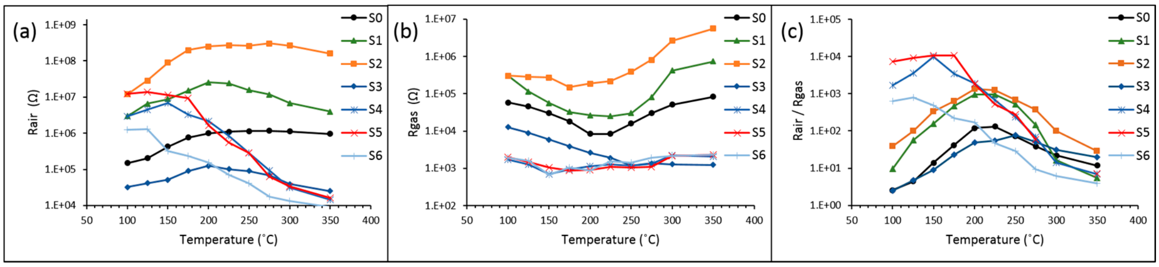

Figure 6. (**a**) Sensors resistance in air under different temperature conditions 100–350 °C; (**b**) resistance of the sensors in the presence of 10 ppm of H_2S as a function of temperature; and (**c**) various responses of sensors toward 10 ppm of H_2S over the temperature range.

Table 3. Optimum operational parameters of sensors S0–S6 upon exposure to 10 ppm of H_2S.

Sample	R Air (Ω)	R Gas (Ω)	R Air/R Gas	Temperature (°C)
S0	1.10×10^6	8.43×10^3	1.31×10^2	225
S1	2.53×10^7	2.64×10^4	9.55×10^2	200
S2	2.50×10^8	1.85×10^5	1.88×10^3	200
S3	8.93×10^4	1.17×10^3	7.60×10^1	250
S4	6.90×10^6	6.99×10^2	9.87×10^3	150
S5	9.39×10^6	8.82×10^2	1.06×10^4	150
S6	3.21×10^5	6.65×10^2	4.83×10^2	150

The content of TiO_2 in the hybrid structure was found to be a crucial parameter that determines sensor performance. Both types of hybrid oxide structures (bilayer and multilayer) demonstrated decline in sensitivity for high contents of TiO_2 in the layer. A thick (20 nm) compact layer of TiO_2 deposited over the SnO_2 layer (S3) affected the layer porosity and caused a decrease in sensor response due to the lack of SnO_2 surface exposure to ambient air even at a higher temperature (300 °C). The bilayer structure of SnO_2 with 20 nm of TiO_2 coating demonstrated very low resistance over the temperature range 100–350 °C, compared to the other bilayer samples with thinner TiO_2 coating. Increasing content of TiO_2 within the multilayer structure (S6) from 10% to 20% decreased the sensitivity of the sensor. The sensor S6 with 20% vol. of TiO_2 demonstrated low resistance over the temperature range 100–350 °C and lower sensitivity compared to the other complex oxides with lower TiO_2 concentration.

Sensors S2 (bilayer) and S5 (multilayer) demonstrated the highest sensitivity to H_2S in their groups due to the optimized content of TiO_2. The bilayer sensor (S2) demonstrated the highest resistance in ambient air among all the sensors (S0–S6), which is an indication of the maximum depletion of carriers in the catalytic layer.

The superior response of the multilayer oxide sensors (S4 and S5) compared to the rest of the sensors, was attributed to the optimal content of TiO_2 uniformly distributed through the volume of the catalytic layers affecting the morphological, electrical and catalytic properties of the sensor. Multilayer structures demonstrated smaller average crystal size after the annealing, higher porosity for 5 and 10% vol. of TiO_2 and the highest surface roughness across all the sensors.

Based on our studies over a wide temperature range, the pure unmodified SnO_2 sensor demonstrated relatively poor H_2S detection capabilities, compared to hybrid (multilayer or bilayer) SnO_2/TiO_2 structures. Also, multilayer structures respond better to hydrogen sulfide exposures, than bilayer structures. We relate this phenomenon to the balance between the catalytic activity of the layer and conversion of this catalytic activity into a measurable signal through the charge transfer. The catalytic activity is determined by the surface area of the interactive layer, grain size and structure and by the number of reaction centers (active sites) in the individual grains. The charge transfer that converts catalytic activity into a measurable signal is determined by the oxygen-induced depletion region underneath the oxide surface and by the multiple heterojunctions between the grains. When these factors are balanced, they amplify each other, which was observed in the multilayer oxide structures. In the bilayer structure, the surface depletion was remarkable (even higher than in the multilayer oxides), but the catalytic activity suffered because of the uncontrolled growth of TiO_2 grains and their agglomeration, which substantially reduced their catalytic activity.

Response and recovery times were found from the sensor response to 10 ppm of H_2S under optimal temperature conditions for each sensor (Table 4). Sensors S2 and S5 demonstrated shortest time for the sensor's response resistance to reach 90% of its steady state value (Figure 7).

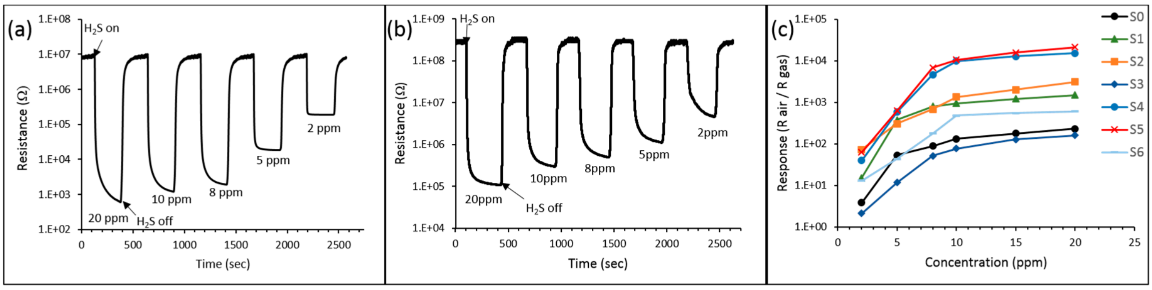

Figure 7. Sensor response of S5 multilayer structure (**a**) and S2 bilayer structure (**b**) to different concentrations of H_2S (from 2 ppm to 20 ppm). Calibration curves Response vs. Concentration for sensors S0–S6 (**c**).

Table 4. Response and recovery time of sensors S0–S6 to 10 ppm of H_2S.

Sample Number	Response Time T_{90} (s)	Recovery Time T_{90} (s)	Concentration (ppm)	Temperature (°C)
S0	3.7	5.6	10	225
S1	3.5	2.8	10	200
S2	3.3	2.5	10	200
S3	3.7	2.9	10	250
S4	3.0	2.4	10	150
S5	3.2	2.4	10	150
S6	3.9	2.7	10	150

Besides excellent sensitivity, quick response and recovery time (Table 4), hybrid sensors also demonstrate superior selectivity to hydrogen sulfide. In our experiments, hybrid sensors were capable of detecting hydrogen sulfide in complex gas mixtures, such as natural gas, which is not typical for metal oxide sensors. The illustration of cross-sensitivity studies on hybrid sensors is shown in the Figure 8. Figure 8a shows the response amplitudes of sensors S5 (multilayer structure) and S2 (bilayer structure) to various gases at different concentrations. Figure 8b shows the response of sensor S5 to sub-ppm concentrations of H_2S diluted in pure methane. We attribute this remarkable selectivity of hybrid layers to high catalytic activity of SnO_2/TiO_2 hybrid structures relative to H_2S at relatively low temperatures. Maximum sensor response for bilayer and multilayer structures was achieved at 200 °C and 150 °C respectively, which is substantially lower than the optimum activation temperature of pure tin dioxide sensor (300 °C). It is related to a lower activation temperature for oxidation of hybrid catalyst, compared to tin dioxide.

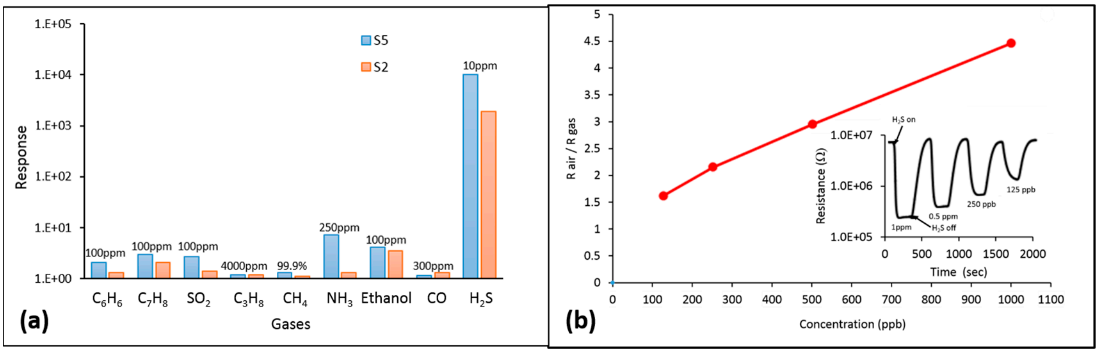

Figure 8. Response amplitudes of sensors S5 (multilayer structure) and S2 (bilayer structure) to various gases (**a**); Response of sensor S5 to sub-ppm concentrations of H_2S diluted in pure methane (**b**).

Because of that, the energy of active sites on the surface is not enough to overcome the activation barrier of combustibles, ethanol and carbon dioxide, which provides a natural cut-off for all the catalytic reactions except for the H_2S decomposition and oxidation.

The major factor that determines chemical sensitivity of a metal oxide sensor is its catalytic activity toward the analyte of interest. Nanoscale titanium dioxide is a very reactive catalyst for the Clauss process and interacts with hydrogen sulfide more efficiently than tin dioxide. Multiple reports show that materials demonstrate the maximum of their catalytic activity in the nanoparticle form, which is related to maximization of the surface area and the number of active sites (reaction centers). In our experiments, both double layer and multilayer-type sensors demonstrated maximum sensitivity at a certain optimum volume percentage of titanium dioxide in the tin dioxide layer. The pure tin dioxide sensor demonstrates moderate sensitivity and no selectivity to hydrogen sulfide. Additionally, a long time of recovery after the exposure is evidence of a relatively low catalytic reaction rate. With an increase of titanium oxide content in the hybrid layer, sensitivity increases dramatically and recovery time drops to a few seconds. Sensors demonstrate the highest sensitivity and the fastest recovery at 10% vol. of titanium oxide. Further increase of titanium oxide content causes the decline of sensor performance, which is associated with agglomeration of titanium nanoparticles into larger grains and reduction of their catalytic activity. Substantial increase in titanium oxide content, overcoming its percolation threshold and formation of a continuous titanium dioxide matrix creates an extremely inert film with no catalytic properties and extremely poor electrical conductivity.

The enhanced sensitivity of a hybrid layer can be better understood considering charge transfer between the grains. The effects of molecular interactions at the surface of metal oxides, and the corresponding changes in their electrical transport properties, has been broadly discussed in literature. A generalized model of sensing is shown in Figure 9. At high temperatures (150 °C–500 °C), intrinsic n-type SnO_2, once exposed to the ambient air, dissociates and ionizes atmospheric oxygen. The process involves adsorption of O_2 molecules, which then trap electrons from the near-surface region of the semiconductor: $O_2 + e^- = O_2^-$ or $O_2 + 2e^- = 2O^-$. The response to hydrogen sulfide arises from its oxidation and the corresponding stripping of O^- from the surface of SnO_2. This, in turn, releases "trapped" electrons back into the bulk. These "released" electrons reduce the width of the depletion layer, concomitant with a reduction in surface band bending, causing an increase in conductance of the SnO_2. Consistent with the n-type properties of SnO_2, the Claus process results in peaks in conductance, which corresponds to electron charge transfer back into the bulk.

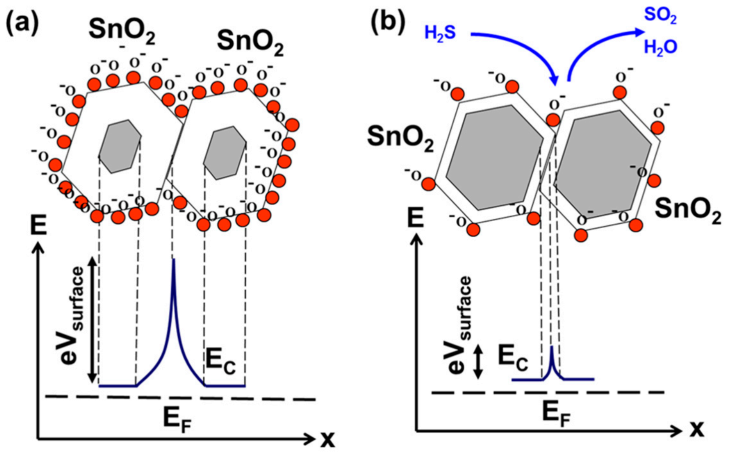

Figure 9. Electronic grain structure of a single-oxide SnO_2 layer in ambient atmosphere (**a**) and upon exposure to hydrogen sulfide (**b**).

The two components of the SnO_2/TiO_2 hybrid layer are both n-type semiconductors, but they differ significantly in their work function and electron affinity. The work function and electron affinity

of TiO_2 are both around 4.2 eV while the work function of SnO_2 is around 4.4 eV and its electron affinity is about 0.5 eV larger than that of TiO_2. The Fermi energy level of TiO_2 is higher than that of SnO_2 because of its smaller work function so electron transfer occurs from the conduction band of TiO_2 to the conduction band of SnO_2. TiO_2 and SnO_2, when crystallized under rutile structure, show very close lattice parameters, favoring the coupling and the heterostructure growth. The formation of the heterojunction and migration of free electrons from the TiO_2 side to the SnO_2 side leads to a discontinuity in the conduction band and formation of the energy barrier at the interface [40].

The effects of charge transfer on chemical sensitivity of a hybrid SnO_2/TiO_2 layer are shown in Figure 10. In the steady state condition, the formation of an electron-enriched zone at the tin dioxide side of the interface enhances oxygen adsorption in this region [18,41]. Additionally, it leads to a more substantial depletion of titanium dioxide grains and formation of contact potential at the SnO_2/TiO_2 boundary. This way the multilayer grain structure of SnO_2/TiO_2 layer amplifies trapping of free electrons at the surface, makes the composite layer depletion more extensive and leads to a larger reduction in conductance due to oxygen adsorption, compared to a single-oxide SnO_2 layer. From our experiments, changes in resistance upon exposure to hydrogen sulfide are much higher for a multilayer heterogeneous system, compared to a single-oxide homogeneous system. This is because in a heterogeneous system more oxygen is available for catalytic surface reactions and more free electrons return back into the bulk during those reactions, than in the homogeneous single-oxide layer. Hence, the changes in resistance upon exposure to hydrogen sulfide are more substantial in the composite layer. Enhanced catalytic activity together with amplified resistance changes due to charge transfer effects make hybrid sensors superior to single-oxide sensors.

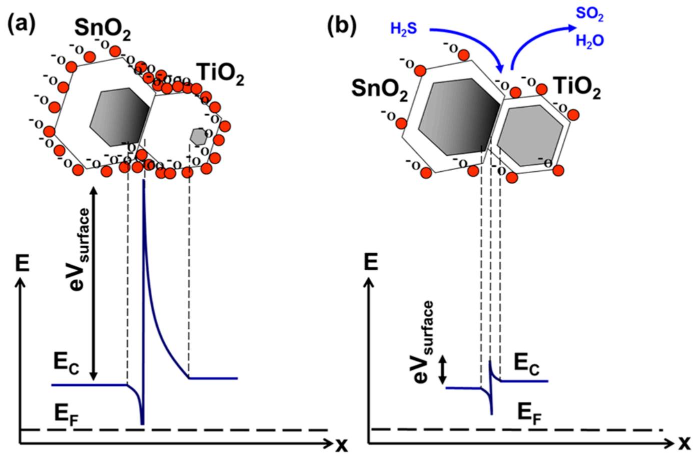

Figure 10. Electronic grain structure of a hybrid SnO_2/TiO_2 layer in ambient atmosphere (**a**) and upon exposure to hydrogen sulfide (**b**).

One of the challenges for metal oxide sensors is the effect of humidity on sensor performance. In our studies, we investigated the operation of sensor S5 under four different humidity levels: 0%, 25%, 50% and 75% (Figure 11). It was found that the humidity level, which is equal or less than 50%, has a minor effect on sensitivity. The total change in sensor response was found to be less than 9% for detection of low H_2S concentration of 2 ppm and less than 12% for higher concentration of H_2S of 20 ppm. Further investigation of sensor S5 performance under higher humidity demonstrated that the sensor sensitivity was drastically affected by humidity higher than 75%. The sensor response decreased for both low (2 ppm) and high (20 ppm) concentrations of H_2S by 33% and 46%, respectively.

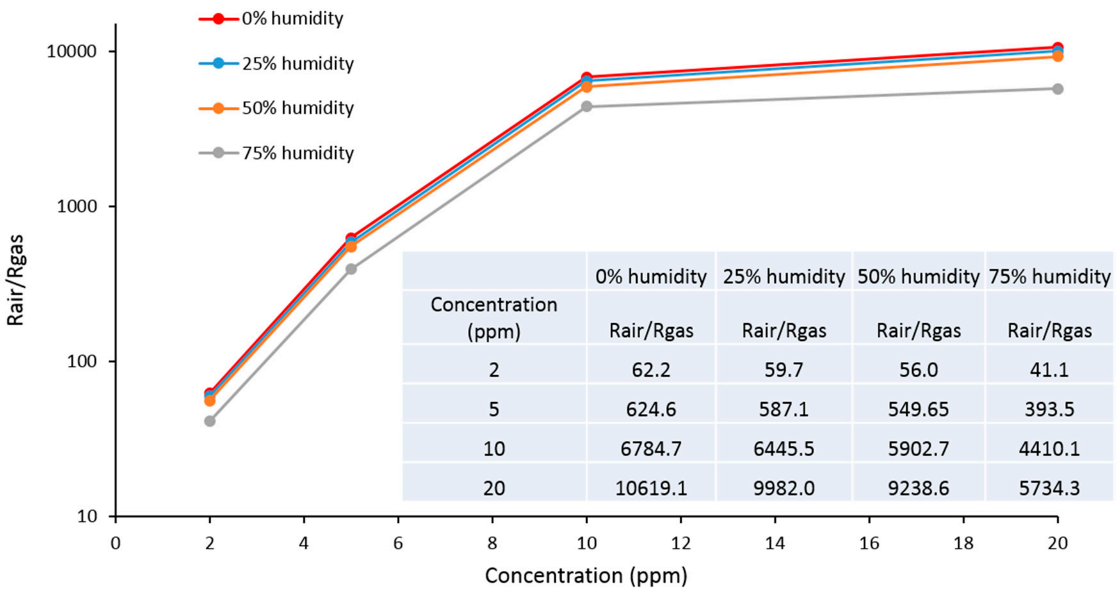

Concentration (ppm)	0% humidity Rair/Rgas	25% humidity Rair/Rgas	50% humidity Rair/Rgas	75% humidity Rair/Rgas
2	62.2	59.7	56.0	41.1
5	624.6	587.1	549.65	393.5
10	6784.7	6445.5	5902.7	4410.1
20	10619.1	9982.0	9238.6	5734.3

Figure 11. Calibration curves Response vs. Concentration for sensors S5 under different humidity levels.

Overall sensor performance within humidity range 0%–50% was found to be better than the performance of commercial MOX sensors (Figaro, MQ, etc.) for a wide range of general purpose sensing applications. Additionally, the sensor humidity error correction for different humidity levels could be obtained for more precise measurements. It is important to notice that even at extremely high levels of humidity higher that 75% the high performance of sensors can be achieved by using an additional inline air desiccant.

4. Conclusions

On the basis of a hybrid SnO_2/TiO_2 structure, a novel metal oxide sensor was developed. Two types of structures were considered: a bilayer structure, where TiO_2 was deposited on top of SnO_2, and a multilayer structure, when six layers of TiO_2 and SnO_2 were deposited one after another in a sequential manner. Both types of sensors were characterized by multiple techniques in order to relate their geometry, morphology and chemical composition to their catalytic activity. It was found that both types of structures show high catalytic activity at relatively low temperatures, when TiO_2 content in SnO_2 is optimized (~10% vol.). Additionally, the optimized hybrid layer demonstrated superior time of response and time of recovery as well as the selectivity to hydrogen sulfide. The multilayer structure demonstrated better operational characteristics than a bilayer structure. The superior performance of the multilayer structure was attributed to the excellent catalytic properties of TiO_2 nanocrystals, coupled with the SnO_2 matrix. By optimizing the hybrid layer, charge transfer between the grains amplifies catalytic activity making the layer extremely sensitive. Additionally, the low activation temperature cuts off most of the undesirable catalytic reactions, making the sensor highly selective. The proposed sensor demonstrates performance superior to sensors from the previously published reports and to the commercially available ones.

Acknowledgments: Funding for this research and publication was provided by VAON LLC.

Author Contributions: Alexander Larin manufactured the sensors, built the calibration rig, assembled the gas delivery system, conducted the sensor surface characterization by different methods and significantly contributed to the article writing. Vladimir Dobrokhotov designed the sensor, conducted electrical characterization of sensors, participated in sensor calibration and testing, formulated theoretical interpretation of the observed results, provided the overall management of the project and significantly contributed to the article writing. Phillip C. Womble was in charge of sensor evaluations for industrial applications and conducting field tests. He developed the list of criteria for sensor optimization in order to achieve the desirable characteristics. Also,

Womble significantly contributed to solving the problem of temperature localization on the metal oxide layers and contributed to the article writing.

Conflicts of Interest: The authors declare no conflict of interest.

References

1. Ou, J.Z.; Ge, W.; Carey, B.; Daeneke, T.; Rotbart, A.; Shan, W.; Wang, Y.; Fu, Z.; Chrimes, A.F.; Wlodarski, W.; et al. Physisorption-Based Charge Transfer in Two-Dimensional SnS_2 for Selective and Reversible NO_2 Gas Sensing. *ACS Nano* **2015**, *9*, 10313–10323. [CrossRef] [PubMed]
2. Mei, L.; Chen, Y.; Ma, J. Gas Sensing of SnO_2 Nanocrystals Revisited: Developing Ultra-Sensitive Sensors for Detecting the H_2S Leakage of Biogas. *Sci. Rep.* **2014**, *4*, 6028. [CrossRef] [PubMed]
3. Verma, M.K.; Gupta, V. A highly sensitive SnO_2–CuO multilayered sensor structure for detection of H_2S gas. *Sens. Actuators B Chem.* **2012**, *166–167*, 378–385. [CrossRef]
4. Wagh, M.S.; Patil, L.A.; Seth, T.; Amalnerkar, D.P. Surface cupricated SnO_2–ZnO thick films as a H_2S gas sensor. *Mater. Chem. Phys.* **2004**, *84*, 228–233. [CrossRef]
5. Xi, L.; Qian, D.; Tang, X.; Chen, C. High surface area SnO_2 nanoparticles: Synthesis and gas sensing properties. *Mater. Chem. Phys.* **2008**, *108*, 232–236. [CrossRef]
6. Hwang, I.-S.; Choi, J.-K.; Kim, S.-J.; Dong, K.-Y.; Kwon, J.-H.; Ju, B.-K.; Lee, J.-H. Enhanced H_2S sensing characteristics of SnO_2 nanowires functionalized with CuO. *Sens. Actuators B Chem.* **2009**, *142*, 105–110. [CrossRef]
7. Chen, J.; Wang, K.; Hartman, L.; Zhou, W. H_2S Detection by Vertically Aligned CuO Nanowire Array Sensors. *J. Phys. Chem. C* **2008**, *112*, 16017–16021. [CrossRef]
8. Kaneti, Y.V.; Yueb, J.; Moriceauc, J.; Chena, C.; Liud, M.; Yuana, Y.; Jiangd, X.; Yua, A. Experimental and theoretical studies on noble metal decorated tin oxide flower-like nanorods with high ethanol sensing performance. *Sens. Actuators B Chem.* **2015**, *219*, 83–93. [CrossRef]
9. Tang, Y.; Su, B.; Liu, M.; Feng, Y.; Jiang, X.; Jiang, L.; Yu, A. Superwettability Strategy: 1D Assembly of Binary Nanoparticles as Gas Sensors. *Small* **2016**. [CrossRef] [PubMed]
10. Yue, J.; Jiang, X.; Yu, A. Adsorption of the OH Group on SnO_2 (110) Oxygen Bridges: A Molecular Dynamics and Density Functional Theory Study. *J. Phys. Chem. C* **2013**, *117*, 9962–9969. [CrossRef]
11. Huang, J.; Wan, Q. Gas Sensors Based on Semiconducting Metal Oxide One-Dimensional Nanostructures. *Sensors* **2009**, *9*, 9903–9924. [CrossRef] [PubMed]
12. Das, S.; Jayaraman, V. SnO_2: A comprehensive review on structures and gas sensors. *Prog. Mater. Sci.* **2014**, *66*, 112–255. [CrossRef]
13. Naik, A.; Parkin, I.; Binions, R. Gas Sensing Studies of an n-n Hetero-Junction Array Based on SnO_2 and ZnO Composites. *Chemosensors* **2016**, *4*, 3. [CrossRef]
14. Yamazoe, N.; Tamaki, J.; Miura, N. Role of hetero-junctions in oxide semiconductor gas sensors. *Mater. Sci. Eng. B* **1996**, *41*, 178–181. [CrossRef]
15. Costello, L.; Ewen, R.J.; Ratcliffe, N.M.; Sivanand, P. Thick film organic vapour sensors based on binary mixtures of metal oxides. *Sens. Actuators B Chem.* **2003**, *92*, 159–166. [CrossRef]
16. Yamazoe, N.; Shimanoe, K. Proposal of contact potential promoted oxide semiconductor gas sensor. *Sens. Actuators B Chem.* **2013**, *187*, 162–167. [CrossRef]
17. Halek, P.; Teterycz, H.; Halek, G.; Suchorska, P.; Wiśniewski, K. Sensing performance of heterojunction gas sensors based on SnO_2, WO_3 and ZnO metal oxides. In Proceedings of the IMCS 2012—The 14th International Meeting on Chemical Sensors, Nuremberg, Germany, 20–23 May 2012; Volume 1297–1300.
18. Suchorska-Woźniak, P.; Rac, O.; Fiedot, M.; Teterycz, H. Analysis of $SnO_2 \mid WO_3$ Heterocontact Properties during the Detection of Hydrogen Sulphide. *Sensors* **2014**, *14*, 20480–20499. [CrossRef] [PubMed]
19. Wager, J.F. Transparent electronics: Schottky barrier and heterojunction considerations. *Thin Solid Films* **2008**, *516*, 1755–1764. [CrossRef]
20. Jiao, Z.; Wang, S.; Bian, L.; Liu, J. Stability of SnO_2/Fe_2O_3 multilayer thin film gas sensor. *Mater. Res.* **2000**, *35*, 741–745. [CrossRef]
21. Guo, W.; Mei, L.; Wenc, J.; Ma, J. High-response H_2S sensor based on ZnO/SnO_2 heterogeneous nanospheres. *RSC Adv.* **2016**, *6*, 15048. [CrossRef]

22. Huang, W.-F.; Chen, H.-T.; Lin, M.C. Density Functional Theory Study of the Adsorption and Reaction of H_2S on TiO_2 Rutile (110) and Anatase (101) Surfaces. *J. Phys. Chem. C* **2009**, *113*, 20411–20420. [CrossRef]
23. Chun, S.W.; Jang, J.Y.; Park, D.W.; Woo, H.C.; Chung, J.S. Selective oxidation of H_2S to elemental sulfur over TiO_2/SiO_2 catalysts. *Appl. Catal. B* **1998**, *6*, 235–243. [CrossRef]
24. Garcia, A.; Yan, N.; Vincent, A.; Singh, A.; Hill, J.M.; Chuang, K.T.; Luo, J.-L. Highly cost-effective and sulfur/coking resistant VO_x-grafted TiO_2 nanoparticles as an efficient anode catalyst for direct conversion of dry sour methane in solid oxide fuel cells. *J. Mater. Chem. A* **2015**, *3*, 23973–23980. [CrossRef]
25. Zhang, X.; Tang, Y.; Qu, S.; Da, J.; Hao, Z. H_2S-Selective Catalytic Oxidation: Catalysts and Processes. *ACS Catal.* **2015**, *5*, 1053–1067. [CrossRef]
26. Radecka, M.; Zakrzewska, K.; Rękas, M. SnO_2–TiO_2 solid solutions for gas sensors. *Sens. Actuators B Chem.* **1998**, *47*, 194–204. [CrossRef]
27. Patil, L.A.; Patil, D.R. Heterocontact type CuO-modified SnO_2 sensor for the detection of a ppm level H_2S gas at room temperature. *Sens. Actuators B Chem.* **2006**, *120*, 316–323. [CrossRef]
28. Vasiliev, R.B.; Rumyantsev, M.N.; Yakovlev, N.V.; Gaskov, A.M. CuO/SnO_2 thin film heterostructures as chemical sensors to H_2S. *Sens. Actuators B Chem.* **1998**, *50*, 186–193. [CrossRef]
29. Chowdhuri, A.; Singh, S.K.; Sreenivas, K.; Gupta, V. Contribution of adsorbed oxygen and interfacial space charge for enhanced response of SnO_2 sensors having CuO catalyst for H_2S gas. *Sens. Actuators B Chem.* **2010**, *145*, 155–166. [CrossRef]
30. Ma, J.; Liu, Y.; Zhang, H.; Ai, P.; Gong, N.; Wu, Y.; Yu, D. Room temperature ppb level H_2S detection of a single Sb-doped SnO_2 nanoribbon device. *Sens. Actuators B Chem.* **2015**, *216*, 72–79. [CrossRef]
31. Yoo, K.S.; Han, S.D.; Moon, H.G.; Yoon, S.-J.; Kang, C.-Y. Highly Sensitive H_2S Sensor Based on the Metal-Catalyzed SnO_2 Nanocolumns Fabricated by Glancing Angle Deposition. *Sensors* **2015**, *15*, 15468–15477. [CrossRef] [PubMed]
32. Yang, T.; Yang, Q.; Xiao, Y.; Sun, P.; Wang, Z.; Gao, Y.; Ma, J.; Sun, Y.; Lu, G. A pulse-driven sensor based on ordered mesoporous Ag_2O/SnO_2 with improved H_2S-sensing performance. *Sens. Actuators B Chem.* **2016**, *228*, 529–538. [CrossRef]
33. Liua, H.; Gonga, S.P.; Hua, Y.X.; Liua, J.Q.; Zhoua, D.X. Properties and mechanism study of SnO_2 nanocrystals for H_2S thick-film sensors. *Sens. Actuators B Chem.* **2009**, *140*, 190–195. [CrossRef]
34. Pedhekar, R.B.; Raghuwanshi, F.C.; Kapse, V.D.; Raisoni, G.H. Low Temperature H_2S Gas Sensor Based on Fe_2O_3 Modified ZnO-TiO_2 Thick Film. *Int. J. Mater. Sci. Eng.* **2015**, *3*, 219. [CrossRef]
35. Vaishampayan, M.V.; Deshmukh, R.G.; Walke, P.; Mulla, I.S. Fe-doped SnO_2 nanomaterial: A low temperature hydrogen sulfide gas sensor. *Mater. Chem. Phys.* **2008**, *109*, 230–234. [CrossRef]
36. Fang, G.; Liu, Z.; Liu, C.; Yao, K. Room temperature H_2S sensing properties and mechanism of CeO_2–SnO_2 sol–gel thin films. *Sens. Actuators B Chem.* **2000**, *66*, 46–48. [CrossRef]
37. Liu, H.; Wu, S.; Gong, S.; Zhao, J.; Liu, J.; Zhou, D. Nanocrystalline In_2O_3–SnO_2 thick films for low-temperature hydrogen sulfide detection. *Ceram. Int.* **2011**, *37*, 1889–1894. [CrossRef]
38. Fu, D.; Zhu, C.; Zhang, X.; Lia, C.; Chen, Y. Two-dimensional net-like SnO_2/ZnO heteronanostructures for high-performance H_2S gas sensor. *J. Mater. Chem. A* **2016**, *4*, 1390. [CrossRef]
39. Tang, H.; Prasad, K.; Sanjinès, R.; Schmid, P.E.; Lévy, F. Electrical and optical properties of TiO_2 anatase thin films. *J. Appl. Phys.* **1994**, *75*, 2042. [CrossRef]
40. Floriano, E.A.; Scalvi, L.V.A.; Saeki, M.J.; Sambrano, J.R. Preparation of TiO_2/SnO_2 Thin Films by Sol−Gel Method and Periodic B3LYP Simulations. *J. Phys. Chem. A* **2014**, *118*, 5857–5865.
41. Trakhtenberg, L.I.; Gerasimov, G.N.; Gromov, V.F.; Belysheva, T.V.; Ilegbusi, O.J. Gas Semiconducting Sensors Based on Metal Oxide Nanocomposites. *J. Mater. Sci. Res.* **2012**, *1*, 56–68. [CrossRef]

© 2016 by the authors; licensee MDPI, Basel, Switzerland. This article is an open access article distributed under the terms and conditions of the Creative Commons Attribution (CC-BY) license (http://creativecommons.org/licenses/by/4.0/).
