# Supplementary material for: Addendum: Larin, A.; Womble, P.C.; Dobrokhotov, V. Hybrid SnO2/TiO2 Nanocomposites for Selective Detection of Ultra-Low Hydrogen Sulfide Concentrations in Complex Backgrounds. Sensors 2016, 16, 1373
Source: Sensors (Basel). 2017 Apr 5;17(4):768. doi: 10.3390/s17040768 (PMC5422041; doi:10.3390/s17040768)
Supplement: Supplementary File 1 [file sensors-17-00768-s001.zip › sensors-16-01373-tex.zip!/sensors-16-01373-g003.pdf]

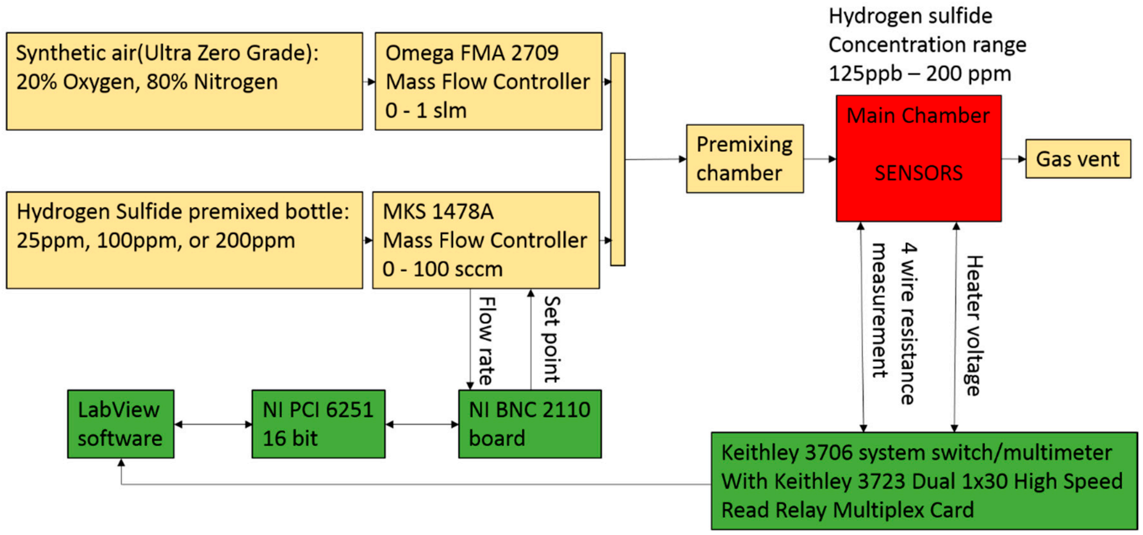
Hydrogen sulfide
Concentration range
125ppb – 200 ppm
Synthetic air(Ultra Zero Grade):
20% Oxygen, 80% Nitrogen
Omega FMA 2709
Mass Flow Controller
0 - 1 slm
Premixing
chamber
Main Chamber
SENSORS
Gas vent
Hydrogen Sulfide premixed bottle:
25ppm, 100ppm, or 200ppm
MKS 1478A
Mass Flow Controller
0 - 100 sccm
Flow rate
Set point
4 wire resistance
measurement
Heater voltage
LabView
software
NI PCI 6251
16 bit
NI BNC 2110
board
Keithley 3706 system switch/multimeter
With Keithley 3723 Dual 1x30 High Speed
Read Relay Multiplex Card
