# Supplementary material for: Addendum: Larin, A.; Womble, P.C.; Dobrokhotov, V. Hybrid SnO2/TiO2 Nanocomposites for Selective Detection of Ultra-Low Hydrogen Sulfide Concentrations in Complex Backgrounds. Sensors 2016, 16, 1373
Source: Sensors (Basel). 2017 Apr 5;17(4):768. doi: 10.3390/s17040768 (PMC5422041; doi:10.3390/s17040768)
Supplement: Supplementary File 1 [file sensors-17-00768-s001.zip › sensors-16-01373-tex.zip!/sensors-16-01373-g004.pdf]

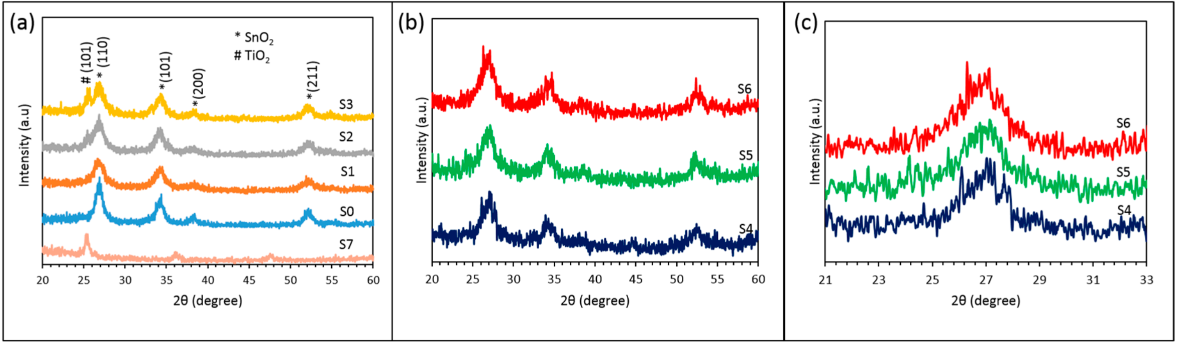

(a)
* SnO_2
# TiO_2
# (101)
* (110)
* (101)
* (200)
* (211)
S3
S2
S1
S0
S7
Intensity (a.u)
2θ (degree)
20 25 30 35 40 45 50 55 60
(b)
S6
S5
S4
Intensity (a.u.)
2θ (degree)
20 25 30 35 40 45 50 55 60
(c)
S6
S5
S4
Intensity (a.u.)
2θ (degree)
21 23 25 27 29 31 33
