# Supplementary material for: Addendum: Larin, A.; Womble, P.C.; Dobrokhotov, V. Hybrid SnO2/TiO2 Nanocomposites for Selective Detection of Ultra-Low Hydrogen Sulfide Concentrations in Complex Backgrounds. Sensors 2016, 16, 1373
Source: Sensors (Basel). 2017 Apr 5;17(4):768. doi: 10.3390/s17040768 (PMC5422041; doi:10.3390/s17040768)
Supplement: Supplementary File 1 [file sensors-17-00768-s001.zip › sensors-16-01373-tex.zip!/sensors-16-01373-g007.pdf]

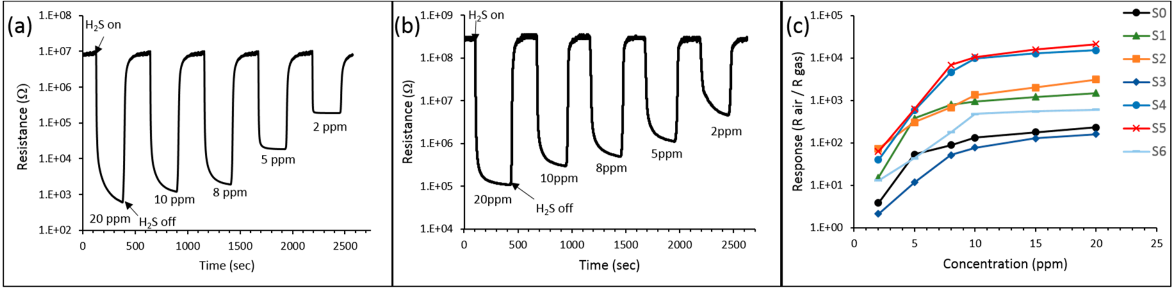
(a)
1.E+08
1.E+07
1.E+06
1.E+05
1.E+04
1.E+03
1.E+02
H_2S on
20 ppm
H_2S off
10 ppm
8 ppm
5 ppm
2 ppm
Resistance (Ω)
0
500
1000
1500
2000
2500
Time (sec)
(b)
1.E+09
1.E+08
1.E+07
1.E+06
1.E+05
1.E+04
H_2S on
20ppm
H_2S off
10ppm
8ppm
5ppm
2ppm
Resistance (Ω)
0
500
1000
1500
2000
2500
Time (sec)
(c)
1.E+05
1.E+04
1.E+03
1.E+02
1.E+01
1.E+00
Response (R air / R gas)
0
5
10
15
20
25
Concentration (ppm)
S0
S1
S2
S3
S4
S5
S6
