# Supplementary material for: Addendum: Larin, A.; Womble, P.C.; Dobrokhotov, V. Hybrid SnO2/TiO2 Nanocomposites for Selective Detection of Ultra-Low Hydrogen Sulfide Concentrations in Complex Backgrounds. Sensors 2016, 16, 1373
Source: Sensors (Basel). 2017 Apr 5;17(4):768. doi: 10.3390/s17040768 (PMC5422041; doi:10.3390/s17040768)
Supplement: Supplementary File 1 [file sensors-17-00768-s001.zip › sensors-16-01373-tex.zip!/sensors-16-01373-g008.pdf]

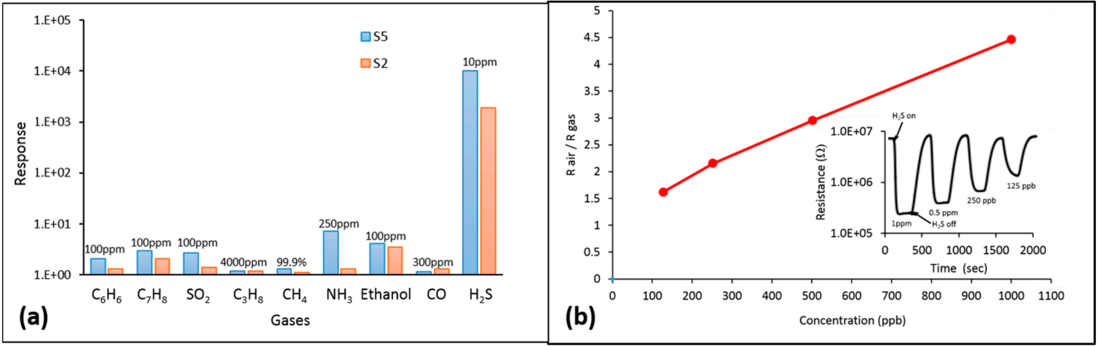

(a)
Response
1.E+05
1.E+04
1.E+03
1.E+02
1.E+01
1.E+00
S5
S2
100ppm
100ppm
100ppm
4000ppm
99.9%
250ppm
100ppm
300ppm
10ppm
C_6H_6
C_7H_8
SO_2
C_3H_8
CH_4
NH_3
Ethanol
CO
H_2S
Gases
(b)
R air / R gas
Concentration (ppb)
0 100 200 300 400 500 600 700 800 900 1000 1100
0 0.5 1 1.5 2 2.5 3 3.5 4 4.5 5
H_2S on
H_2S off
Resistance (Ω)
1.0E+07
1.0E+06
1.0E+05
1ppm
0.5 ppm
250 ppb
125 ppb
Time (sec)
0 500 1000 1500 2000
