# Supplementary material for: Addendum: Larin, A.; Womble, P.C.; Dobrokhotov, V. Hybrid SnO2/TiO2 Nanocomposites for Selective Detection of Ultra-Low Hydrogen Sulfide Concentrations in Complex Backgrounds. Sensors 2016, 16, 1373
Source: Sensors (Basel). 2017 Apr 5;17(4):768. doi: 10.3390/s17040768 (PMC5422041; doi:10.3390/s17040768)
Supplement: Supplementary File 1 [file sensors-17-00768-s001.zip › sensors-16-01373-tex.zip!/sensors-16-01373-g011.pdf]

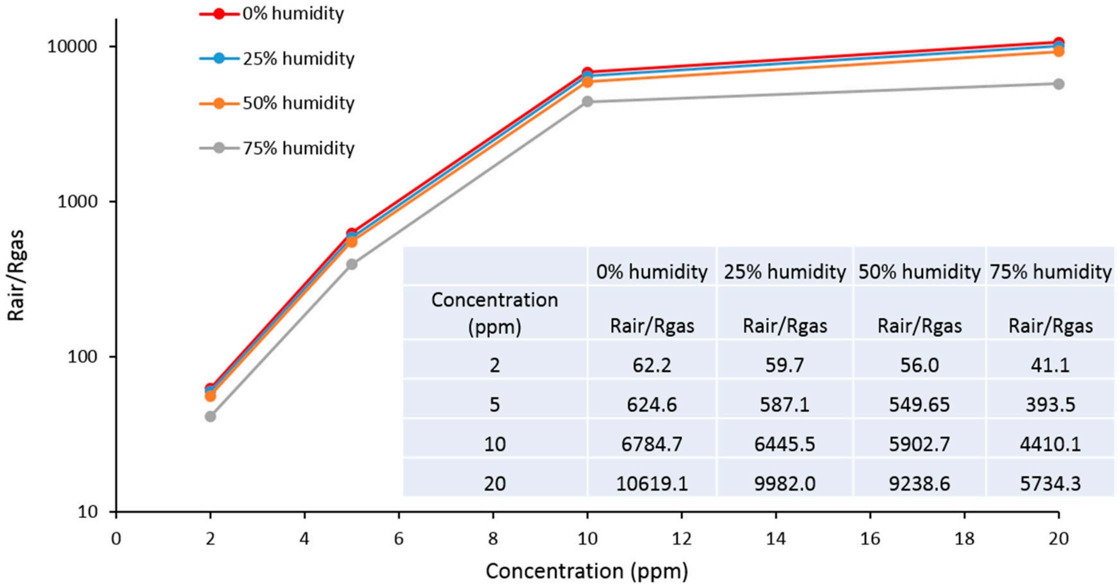

	0% humidity	25% humidity	50% humidity	75% humidity
Concentration (ppm)	Rair/Rgas	Rair/Rgas	Rair/Rgas	Rair/Rgas
2	62.2	59.7	56.0	41.1
5	624.6	587.1	549.65	393.5
10	6784.7	6445.5	5902.7	4410.1
20	10619.1	9982.0	9238.6	5734.3
