# Supplementary material for: Addendum: Larin, A.; Womble, P.C.; Dobrokhotov, V. Hybrid SnO2/TiO2 Nanocomposites for Selective Detection of Ultra-Low Hydrogen Sulfide Concentrations in Complex Backgrounds. Sensors 2016, 16, 1373
Source: Sensors (Basel). 2017 Apr 5;17(4):768. doi: 10.3390/s17040768 (PMC5422041; doi:10.3390/s17040768)
Supplement: Supplementary File 1 [file sensors-17-00768-s001.zip › sensors-16-01373-tex.zip!/logo-ccby-eps-converted-to.pdf]

cc
BY
